# Instruments for measuring nursing research competence: a COSMIN-based scoping review

**DOI:** 10.1186/s12912-023-01572-7

**Published:** 2023-10-31

**Authors:** Yuting Xia, Hui Huang, Xirongguli Halili, Siyuan Tang, Qirong Chen

**Affiliations:** 1 Xiangya School of Nursing, Central South University, 172 Tongzipo Road, Changsha, 410000 Hunan China; 2 The Third Xiangya Hospital, Central South University, Changsha, China; 3 Xiangya Research Center of Evidence-Based Healthcare, Central South University, Changsha, China

**Keywords:** Instruments, Nursing research competence, Scoping review, COnsensus-based Standards for the selection of health Measurement Instruments, COSMIN

## Abstract

**Aim:**

The aim of this scoping review was to evaluate and summarise the measurement properties of nursing research competence instruments and provide a summary overview of the use of nursing research competence instruments.

**Background:**

Increasing nursing research competence instruments have been developed. However, a systematic review and evaluation of nursing research competence instruments is lacking.

**Method:**

This scoping review was conducted following the Joanna Briggs Institute updated methodology for scoping reviews and reported according to the Preferred Reporting Items for Systematic Reviews and Meta-Analyses extension for Scoping Reviews checklist. Reviewers searched articles in Eight English databases and two Chinese databases between April 1st, 2022, and April 30th, 2022. An updated literature search was conducted between March 1st and March 4th, 2023. The literature screening and data extraction were conducted by two reviewers, independently. A third reviewer was involved when consensus was needed. The COnsensus-based Standards for the selection of health Measurement Instruments methodology was used to evaluate the methodological quality and measurement properties of the nursing research competence instruments.

**Results:**

Ten studies involving eight nursing research competence instruments were included. None of the existing instruments have assessed all measurement properties. A total of 177 empirical studies have utilized a nursing research competence instrument with tested measurement properties.

**Conclusion:**

‘Self-evaluated Nursing Research Capacity of Questionnaire (refined)’ was identified as the most appropriate nursing research competence instrument in existing instruments. However, reviewers need to conduct further measurement properties studies on the existing nursing research competence instruments.

**Implications for the nursing policy:**

This study could guide the selection of appropriate nursing research competence instruments which could help to evaluate the nursing research competence of nurses and inform the development of intervention plans to enhance nursing research competence.

**Supplementary Information:**

The online version contains supplementary material available at 10.1186/s12912-023-01572-7.

## Introduction

Nursing research competence (NRC) refers to the individual nurse’s ability to conduct nursing research activities [1, 2]. Evidence-based nursing has developed rapidly in recent years, and the importance of evidence-based nursing in improving clinical nursing quality has been confirmed by many researchers [3–5]. However, there is currently a lack of relevant available evidence focusing on clinical problems, so it is necessary for some nurses with nursing research competence to conduct original research on clinial practice in order to generate relevant available evidence and promote evidence-based nursing practices [6]. Specifically, enhancing the NRC of nurses holds significant importance in the advancement of high-quality clinical nursing research. For clinical nurses who are inclined towards research, possessing a strong NRC competence can motivate them to address clinical issues scientifically, apply evidence-based practices, and contribute to bridging the gap between theory and practical application [7]. As future nursing researchers and nurses, improving the NRC of nursing students has a positive promoting effect on the future development of nursing [8, 9]. Using NRC instruments are necessary to evaluate the NRC of nursing staff and the effectiveness of interventions [8, 10].

Measuring the NRC of nursing staff is important for research, education, and management purposes. Research has shown that clinical nurses are the end users and producers of nursing research, and nurses with research competence can promote the development of nursing discipline [11]. The prerequisite for improving nurses' research competence is to clarify the current situation and influencing factors of nurses' research competence, which provides a precise theoretical basis for formulating intervention plans to improve nursing staff's research competence [11]. However, an important way to clarify the current state of NRC and its associated factors was to use precise NRC instruments to measure NRC. They can provide evidence for building effective intervention strategies in research, evaluating teaching quality and promote the development of courses or training programs in education [9]. In addition, using the NRC instruments to measure the NRC of nurses could help nursing managers identify which nurses have good research competence, assist in organizing and conducting research projects, and cultivate research-oriented nurses in a targeted manner [10, 12]. Therefore, it is important to evaluate the measurement properties and the application of existing NRC instruments. This could aid in selecting the most appropriate instrument and in revising or/and developing higher-quality instruments. COSMIN (Consensus-based Standards for the Selection of Health Measurement Instruments) is a consensus-based standard for the selection of health measurement instruments, which can evaluate the methodological quality and measurement properties of measuring instruments and provide recommendations for instrument selection [13]. This study evaluated all measurement properties of the NRC instruments based on COSMIN methodology. For more detailed steps on COSMIN methodology were showed in the 'Methods' section.

### Literature review

Recently, many NRC instruments have been developed, such as the Self-evaluated Nursing Research Capacity Questionnaire for nursing staff by Liu [14], later refined by Pan [15], the Research Competence Scale for nursing students by Qiu [9], and the Scientific Research Competency Scale for nursing professionals at the undergraduate and graduate levels by Pinar Duru [16]. However, researchers are unsure about how to accurately choose an instrument to measure the NRC of the target population. The selection of instrument directly affects the accuracy and credibility of empirical research results.

Research performed with outcome measurement instruments of poor or unknown quality constitutes a waste of resources and is unethical [13]. Selecting a measurement instrument with good reliability and validity is crucial to accurately evaluate NRC. While there are numerous instruments available for measuring NRC [9, 15–17], to our knowledge there is still a lack of comprehensive evaluation and research on the selection and development of guiding NRC instruments [8]. Therefore, the purpose of this scoping review is to identify, evaluate, compare, and summarize the current NRC instruments and their usage, to provide guidance for researchers in selecting appropriate NRC instruments and developing new ones in the future.

This scoping review could answer the following questions [8]: (1) Which NRC instruments have been developed and how they were used in related studies? (2) Were there any well-validated and reliable instruments for measuring NRC? (3) If there were more than one well-validated and reliable instrument for measuring NRC, were there circumstances under which certain instruments were more appropriate for measuring NRC than the other instruments? (4) What were the differences between NRC instruments designed for different groups (e.g., clinical nurses, nursing students)? and (5) What were potential directions for the future development and improvement of NRC instruments?

## Methods

### Objectives

- (1) To identity, evaluate, compare, and summarize the validated instruments developed to measure nursing research competence.
- (2) To provide an overview of the use of all NRC instruments.

### Protocol and registration

This scoping review was conducted following: (1) the Consensus-based Standards for the Selection of Health Measurement Instruments (COSMIN) guidance [13], (2) Joanna Briggs Institute (JBI) updated methodology for scoping review [18] and was reported following the Preferred Reporting Items for Systematic Reviews and Meta-Analyses extension for Scoping Reviews checklist (PRISMA-ScR checklist) [19]. A protocol for this scoping review had been published [8] and registered on the Open Science Framework (osf.io/ksh43).

### Search strategy

Reviewers searched for articles in eight English databases, including the Cochrane Library, Cumulative Index to Nursing and Allied Health Literature (CINAHL), Excerpta Medica Database (EMBASE), PubMed, PsycINFO, Scopus, Education Resource Information Center (ERIC), and ProQuest Dissertations & Theses Global as well as two Chinese databases, namely the China National Knowledge Infrastructure (CNKI) and WANGFANG DATA between April 1st, 2022, and April 30th, 2022. An updated literature search was conducted between March 1st and March 4th, 2023, covering the literature during the period from April 1st, 2022, to March 1st, 2023. Our search methodology was guided by the COSMIN guideline. It encompassed three primary components: (1) the target demographic (e.g., nurses, nursing students), (2) the focal concept (e.g., research, competence), and (3) the measurement attributes (e.g., internal consistency, content validity, among others). Elaborate search strategies for each database could be found in Tables Supplementary 4–13 (Tables S4-S13) within the supplementary material.

### Eligibility criteria

The scoping review aimed to (1) summarize the instruments developed to measure NRC and (2) provide an overview of their use [8]. The inclusion criteria were as follows: (1) the instruments aim to measure NRC; (2) studies that targeted various nursing personnel (e.g., nurses, nursing students, nursing teachers et al.); (3) studies should concern NRC instruments; (4) the aim of the study should be the evaluation of one or more measurement properties, the development of NRC instruments, or the evaluation of the interpretability of the NRC (5) studies that published between 1999 and 2023 (We selected a time frame limit based on most of the research related to NRC were published after 1999, and our search conducted between April 1st, 2022, and April 30th, 2022. An updated literature search was conducted between March 1st and March 4th, 2023, covering the literature during the period from April 1st, 2022, to March 1st, 2023.); (6) studies had available full-text*.*

### Study screening

All studies were exported to an EndNote X9 library (Clarivate Analytics, USA), and duplicates were removed using its deduplication function. Two reviewers (YX and HH) independently screened the titles and abstracts, followed by assessment of full tests of potentially eligible articles. Disagreements between the two reviewers were resolved by a third reviewer (QC). Any articles that were not available online or through author contact were excluded, and The references of the included studies were also screened using the same process.

### Data extraction

Two reviewers (YX and HH) independently extracted data from Tables S1-S4 in the published protocol of this scoping review [20]. A third reviewer (QC) reviewed the results and any disagreements were solved by discussion.

For all eligible studies of objective (1), we extracted information including the development and verification of instruments, measurement properties of the included the development and verification of instrument. However, none of the self-designed scales provided details about the development of NRC instruments or psychometric testing. Furthermore, the evaluation of these scales did not adhere to the COSMIN methodology nor was their data extracted in this study. The extracted data are shown in Table S1, Table S2, and Table 1.

**Table 1:** The methodological evaluation results of studies on measurement properties of NRC instruments

Instrument	Reference	Content validity	Structural validity	Internal consistency	Cross-cultural validity	Reliability	Measurement error	Criterion validity	Hypothesis testing for construct validity	Responsiveness
The Student Research Competence Instrument ①	(Arthur & Wong, 2000)	—	—	very good	—	doubtful	—	—	—	—
The Nursing Research Questionnaire among Nurse Clinicians ②	(Gething et al. 2001)	inadequate	—	very good	—	—	—	—	—	—
The Research Competency Scale for Nursing Students ③	(Qiu et al. 2019)	inadequate	very good	doubtful	—	doubtful	—	—	—	—
Scientific Research Competency Scale ④	(Duru & Örsal, 2021)	doubtful	adequate	very good	—	doubtful	—	—	(1) doubtful (2) doubtful	—
Self-evaluated Nursing Research Capacity Questionnaire ⑤	(Liu, 2004)	doubtful	adequate	very good	—	doubtful	—	—	—	—
The Nursing Scientific Research Ability Scale ⑥	(Wu et al. 2016)	inadequate	adequate	very good	—	doubtful	—	—	—	—
Self-evaluated Nursing Research Capacity Questionnaire (refined) ⑦	(Pan, 2011)	doubtful	very good	very good	—	doubtful	—	—	—	—
	(Chu et al. 2013)	doubtful	very good	very good	—	doubtful	—	—	—	—
Research Capacity Self-rating Scales of Nursing Staff ⑧	(Yin et al. 2016)	doubtful	adequate	very good	—	doubtful	—	—	doubtful	—

^1^ The symbol “—” indicates that the measurement property was not reported in the article and the author cannot be reached for further information

For all eligible studies of objective (2), we extracted the information including author, year, location, study aim, design (intervention), participants, sample size, the instrument of NRC used, and results related to NRC. The information is shown in Table S3 in the supplementary file.

### Quality appraisal and data synthesis

Two reviewers (YX and HH) appraised the quality of the studies, with a third reviewer (QC) resolving any disagreement. First, the content validity (instrument development and content validity) was considered the most important section to determine whether the instrument items were suitable for the construct of interest and target population. Next, evaluating the internal structure (structural validity, internal consistency, and cross-cultural validity) was crucial to understand how the items were combined into a scale or subscale. Finally, the remaining measurement properties (reliability, measurement error, criterion validity, hypotheses testing for construct validity, and responsiveness) were also taken into account [13]. Based on the COSMIN methodology, the studies for objective (1) were evaluated through the following three sections.

### Evaluation of methodological quality

COSMIN Risk of Bias Checklist was used to evaluate the risk of bias of 10 measurement properties (including content validity, structural validity, internal consistency, cross-cultural validity, reliability, measurement error, criterion validity, construct validity hypothesis testing, and responsiveness) [13]. The COSMIN Risk of Bias Checklist has 116 items, each item has five options, including “very good”, “adequate”, “doubtful”, “inadequate”, and “not applicable”. The overall rating of the quality of each study on every measurement properties was determined using the lowest rating among the items [13] (Table 1).

### Evaluation the quality of measurement properties

The methodological quality ratings of instrument development and reviewer's ratings were used to evaluate content validity against the 10 Criteria for Good Content Validity, scoring each measure as "sufficient ( +)", "insufficient (-)", or "inconsistent ( ±)" [13]. The overall rating for a measure was determined by the ratings for relevance, consistency, and comprehensiveness, with inconsistent ratings being scored as ( ±) [13] (Table 2). The results of other psychometric property (including structural validity, internal consistency, cross-cultural validity, reliability, measurement error, criterion validity, construct validity hypothesis testing, and responsiveness) were evaluated against updated criteria for good measurement properties, and were rated as "sufficient ( +)", "insufficient (-)", or "indeterminate (?)" [13] (Table 2). The overall rating was based on the synthesized results, and the synthesized results were generated based on the measurement properities of each single study.

**Table 2:** Overall evaluation results for the measurement properties of NRC instruments and recommendation result

**Instrument**	**Content validity**		**Structural validity**		**Internal consistency**		**Cross-cultural validity**				
	**Overall rating**	**Quality of evidence**	**Overall rating**	**Quality of evidence**	**Overall rating**	**Quality of evidence**	**Overall rating**				**Quality of evidence**
The Student Research Competence Instrument ①	inconsistent	very low	—	—	insufficient	moderate	—^1^				—
The Nursing Research Questionnaire among Nurse Clinicians ②	inconsistent	very low	—	—	sufficient	moderate	—				—
The Research Competency Scale for Nursing Students ③	inconsistent	very low	sufficient	high	insufficient	low	—				—
Scientific Research Competency Scale ④	inconsistent	very low	indeterminate	high	sufficient	high	—				—
Self-evaluated Nursing Research Capacity Questionnaire ⑤	sufficient	moderate	indeterminate	high	indeterminate	high	—				—
The Nursing Scientific Research Ability Scale ⑥	inconsistent	very low	indeterminate	high	sufficient	high	—				—
Self-evaluated Nursing Research Capacity Questionnaire (refined) ⑦	sufficient	moderate	sufficient	high	indeterminate	high	—				—
Research Capacity Self-rating Scales of Nursing Staff ⑧	sufficient	moderate	indeterminate	high	indeterminate	high	—				—
**Instrument**	**Reliability**		**Measurement error**		**Criterion validity**		**Hypothesis testing for construct validity**		**Responsiveness**		**Recommendation**
	**Overall rating**	**Quality of evidence**	**Overall rating**	**Quality of evidence**	**Overall rating**	**Quality of evidence**	**Overall rating**	**Quality of evidence**	**Overall rating**	**Quality of evidence**	
The Student Research Competence Instrument ①	indeterminate	very low	—	—	—	—	—	—		—	B
The Nursing Research Questionnaire among Nurse Clinicians ②	—	—	—	—	—	—	—	—		—	B
The Research Competency Scale for Nursing Students ③	indeterminate	low	—	—	—	—	—	—		—	B
Scientific Research Competency Scale ④	indeterminate	low	—	—	—	—	(1) sufficient (2) sufficient	low low		—	B
Self-evaluated Nursing Research Capacity Questionnaire ⑤	indeterminate	low	—	—	—	—	—	—		—	B
The Nursing Scientific Research Ability Scale ⑥	indeterminate	low	—	—	—	—	—	—		—	B
Self-evaluated Nursing Research Capacity Questionnaire (refined) ⑦	indeterminate	low	—	—	—	—	—	—		—	B
Research Capacity Self-rating Scales of Nursing Staff ⑧	indeterminate	low	—	—	—	—	sufficient	low		—	B

^1^ The symbol “—” indicates that the measurement property was not reported in the article and the author cannot be reached for further information

### Grading of the evidence

The modified GRADE approach was used to rate the quality of evidence, based on the number and quality of available studies, their results, reviewer ratings, and consistency of results. The overall quality was graded as "High", "Moderate", "Low", or "Very low" [13]. Evidence quality was further downgraded based on the presence of risk of bias, inconsistency, and indirectness [13] (Table 2).

Studies that only used the NRC instrument as a variable without testing its properties would not be evaluated, but their characteristics would be extracted.

### Recommendation

Instruments were categorized using COSMIN guidelines into three groups: (A) Instruments with evidence for sufficient content validity (any level) AND at least low quality evidence for sufficient internal consistency; (B) Instruments categorized not in A or C; (C) Instruments with high quality evidence for any an insufficient measurement property [13].

Instruments categorized as (A) could be recommended for widely use. Instruments categorized as (B) have potential to be recommended for use, but further research was needed to assess the quality of this instrument. Instruments categorized as (C) should not be recommended for use.

## Results

### Search results

**Fig. 1:**
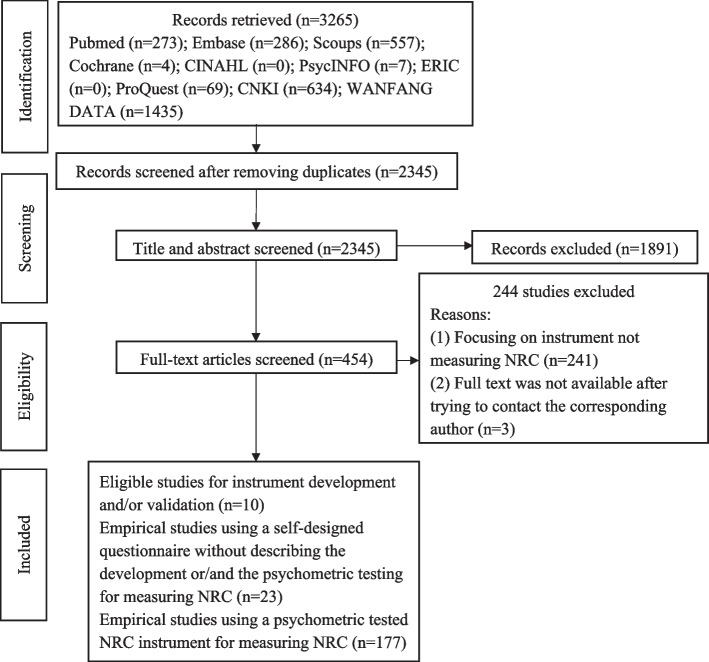
PRISMA flow diagram for this scoping review

A total of 3265 articles were retrieved, 920 duplicates were removed, and 454 were screened for eligibility. From these, 10 studies on NRC instrument development and psychometric properties, 177 empirical studies using a psychometric tested NRC instrument, and 23 empirical studies using a self-designed NRC questionnaire (without describing the development or/and the psychometric testing) were identified (Fig. 1).

### Study characteristics

Tables S1 and S2 presents characteristics of eligible NRC instruments and study populations for objective (1). Six original instruments [9, 14, 16, 21–23], two modified instruments [10, 15, 17], and one psychometric property testing of one NRC instrument are featured in these tables [24]. However, among the ten articles, two articles (one dissertation and another published in a peer-reviewed journal) were published by the same author describing the same instrument [10, 15]. Therefore, we only extracted and evaluated data from the dissertation for this instrument [15]. Self-designed scales without description of the development or psychometric testing were not included in the quality appraisal.

Table S3 shows an overview of all eligible studies for objective (2), along with the NRC instruments that were identified and the number of studies that utilized each specific instrument. The NRC instrument ⑦ adapted by Pan was the most commonly used instrument, with a frequency of 127 [15]. The NRC instrument ⑤ developed by Liu was used 38 times to measure the NRC of nurse staff [14]. The NRC instrument ⑧ was used seven times, and the NRC instrument ③ was used twice. The NRC instruments ② and ⑥ were both used only once to measure the NRC. However, the NRC instruments ① and ④ have not been used. Self-designed NRC instruments without validation were used in 23 studies.

### The results of NRC instruments evaluation

#### Evaluation of methodological quality

The result of methodological quality showed in Table 1. Among the nine studies included, none evaluated all measurement attributes [9, 14–17, 21–24]. Cross-cultural validity, measurement error, criterion validity, and responsiveness have not been evaluated in any of the studies.

### Evaluation the quality of measurement properties

The content validity ratings for five NRC instruments (①②③④⑥) was rated as 'inconsistent ( ±)' and NRC instruments (⑤⑦⑧) was rated as ‘sufficient ( +)’. The assessment of structural validity for two NRC instruments (③⑦) was rated as 'sufficient ( +)’, the assessment of the structural validity of these four NRC instruments (④⑤⑥⑧) was ‘indeterminate (?)’. The assessment of internal consistency for three NRC instruments (②④⑥) was rated as ' sufficient ( +)', two NRC instruments (①③) was rated as ‘insufficient (-)’, and three NRC instruments (⑤⑦⑧) was rated as ‘indeterminate (?)’. The measurement properties of reliability for seven NRC instruments (①③④⑤⑥⑦⑧) was rated as ‘indeterminate (?)’. The assessment of the hypotheses testing for construct validity for NRC instruments ④ and ⑧ was rated as 'sufficient ( +)'. More details were shown in Table 2.

### Grading of the evidence

As present in Table 2, NRC instruments ⑤⑦⑧ was rated as ‘moderate’ for content validity, other NRC instruments (①②③④⑥) were rated as ‘very low’. The quality of the evidence for structural validity of six NRC instruments (③④⑥⑤⑦⑧) were rated as ‘high’. The evidence quality for internal consistency of NRC instruments ① and ② was rated as ‘moderate’, while NRC instrument ③ was rated as ‘low’. NRC instruments ④⑤⑥⑦⑧ was rated as ‘high’ in terms of internal consistency. The evidence quality for the hypotheses testing for construct validity was rated as 'low' for NRC instruments ④ and ⑧.

### Recommended NRC instruments

Based on the evaluation results, three NRC instruments (⑤⑦⑧) were rated as ‘sufficient ( +)’ for content validity, but their internal consistency was rated as ‘indeterminate (?)’ (Table 2). Thus, they were recommended for use under category B. The other NRC instruments (①②③④⑥) had evidence of ‘indeterminate ( ±)’ content validity and lacked high-quality evidence indicating that their content validity was ‘insufficient’ (Table 2). Therefore, these instruments (①②③④⑥) were also recommended for use under category B (Table 2). As all NRC instruments (①②③④⑤⑥⑦⑧) were recommended for use under category B, which mean these NRC instruments (①②③④⑤⑥⑦⑧) have the potential to be recommended, but further validation studies were needed [13].

### The overview of the usage of all NRC instruments

Most studies on NRC instruments were conducted in China (197/200, 98.5%), with cross-sectional studies (147/200, 73.5%), randomized controlled trials (18/200, 9%), a quasi-experimental study (1/200, 0.5%) and before-after studies being the predominant study designs (34/200, 17%). All the studies (100%) were published after 2009, with most studies targeting either nurses (121/200, 60.5%) or nursing students (66/200, 33%) as the target population. Further details on objective (2) can be found in Table S3.

## Discussion

This scoping review evaluated eight NRC instruments using the COSMIN checklist, but none of them have assessed all measurement properties. Among the existing eight NRC instruments, NRC instrument ⑦ is the most widely used, and it has only been used by Chinese scholars as of now. This may be because the NRC instrument ⑦ was developed by Chinese scholar Pan et al. In addition, NRC instrument ⑦ was developed in 2011 and was one of the earliest NRC instruments developed in China.

Lack of reference to the target population during development was an important disadvantage in developing NRC instruments. The items in NRC instruments should be both relevant and comprehensive for the "construct" being measured, as well as comprehensible for the study population. These elements are crucial for ensuring content validity, which is crucial for ensuring an instrument's psychometric properties, and? requires cognitive interviews with the target population [13, 20]. However, only two NRC instruments (⑦⑧) conducted cognitive interviews with the target population during development, and these interviews lacked detail. However, details of the cognitive interview process were missing. Additionally, three studies (⑤⑦⑧) asked the target population about the relevance, comprehensiveness, and comprehensibility of the instrument's content validity, while experts were consulted about the relevance and comprehensiveness of the instruments in all three studies. Comprehensive details of the cognitive interview process are necessary to evaluate content validity. However, the published articles lacked such details, which may be due to the COSMIN guideline being published in 2018, while most (75%) NRC instruments in this review were developed prior to 2018 [25].

Confirmatory factor analysis (CFA) and exploratory factor analysis (EFA) were performed on six NRC instruments (③④⑤⑥⑦⑧), with two instruments (③⑦) reporting CFI values of 0.98 and 0.97, respectively. These NRC instruments (③④⑤⑥⑦⑧) are capable of reliably capturing the theoretical structure and idiosyncratic degree [26]. In other words, these NRC instruments (③④⑤⑥⑦⑧) could effectively represent the theoretical concept of nursing research competence.

Most studies focused on internal consistency, which reflects the correlation of items in the NRC instrument or subscales. However, some NRC instruments ①②④⑤⑥ and ⑧ did not meet the criterion for sufficient structural validity [13]. Therefore, reviewers should evaluate the structural validity before assessing internal consistency and provide detailed information in the future. In addition, NRC instrument ③ only reported Cronbach's alpha for the total instrument, whereas in future studies, reliability analysis should be conducted to evaluate Cronbach's alpha for each dimension of NRC instrument ③. It is worth noting that the Cronbach's alpha values of three subscales in NRC instrument ⑦ were below 0.70 (0.68, 0.68, 0.66, respectively). The value of Cronbach's alpha is influenced by factors such as the number of items, item interrelatedness, and dimensionality [27]. The low Cronbach's alpha value suggests that heterogeneity exists between some items of the instrument and that these items should be revised or removed. One straightforward method is to calculate the item-total score correlation and eliminate items with low correlations [27, 28]. Therefore, additional studies are necessary to enhance and assess the internal consistency of NRC instrument ⑦. Moreover, the sample sizes of studies assessing the internal consistency of NRC instruments ① and ② were below 100, resulting in downgrading of the quality of evidence on internal consistency. Consequently, a larger target population is required to further evaluate the internal consistency of these two NRC instruments (①②).

The prerequisite for widespread use of NRC instruments is to ensure reliability, minimal measurement error, and sensitivity to changes. Except 'The Nurse Research Questionnaire among Nurse Clinicians ②', all NRC instruments have been evaluated for reliability. The reported ICC values were not clearly documented in the literature, indicating that their reliability was not satisfactory. Although reliability and measurement error are interrelated measurement properties [25], there is currently no NRC instrument available to evaluate measurement error. Measurement error refers to the systematic and random errors in the target population's rating, which are not attributed to actual changes in the structure to be measured [27]. However, the credibility of the results obtained from the NRC instruments evaluated in this study may be compromised by the lack of measurement error assessment. Therefore, future research should address this issue by evaluating the measurement error of NRC instruments. Moreover, none of the NRC instruments were tested for responsiveness, which may be attributed to the lack of longitudinal validation, including intervention studies [29]. Although NRC instruments have been utilized in some intervention studies to evaluate outcomes, the minimal important change/distribution of scores (MIC/SD) change score for the stable group target was not calculated [13]. Future research should include more longitudinal or intervention studies that employ NRC instruments to assess their reliability and responsiveness [25].

Criterion validity of NRC instruments ④ and ⑧ was reported using the author-defined gold standard in the respective articles. However, we question the appropriateness of using 'The Anxiety Scale Towards Research and the Attention Scale Towards Scientific Research' and 'General Self Efficiency Scale' as the gold standard in the studies, as they may not be ideal measures for NRC instruments ④ and ⑧, respectively [13]. According to the guidance of the COSMIN guideline, the criterion validity reported in the articles for NRC instruments ④ and ⑧ would be more appropriately considered as convergent validity, which pertains to the hypothesis of the targeted instrument's relationship with other relevant measurement instruments [30]. As a result, we opted not to evaluate criterion validity of NRC instruments ④ and ⑧, and instead focused on testing hypotheses for construct validity (specifically, convergent validity). The challenge of identifying a suitable 'gold standard' for NRC instruments may be attributed to the difficulty in establishing an objective index for NRC. To address this issue, we recommend enhancing the development of objective evaluation indicators for NRC, which could lead to the formation of a 'gold standard' instrument. Having a reliable gold standard could aid in the development and validation of more user-friendly and efficient NRC instruments.

Hypotheses testing for construct validity is defined as the relationships of scores on the instrument of interest with the scores on other instruments measuring similar constructs (convergent validity) or dissimilar constructs (discriminant validity), or the difference in the instrument scores between subgroups of people (known-groups validity) [31]. The study on NRC instrument ④ reported hypotheses stating that individuals with high levels of research competency would hold more positive attitudes towards scientific research and experience less anxiety towards research [16]. Although the hypothesis was not explicitly stated in the study on NRC instrument ⑧, the positive correlation observed between NRC and general self-efficiency could be used to draw conclusions about the construct validity of NRC instrument ⑧ [13, 17]. The studies on NRC instruments ④ and ⑧ all formulated hypotheses for testing construct validity, with expected directions of effect. To accurately represent the underlying theoretical structure of nursing research competence, hypotheses should verify both the magnitude of correlations or differences [31].

No studies evaluated the cross-cultural validity of the NRC instruments. Cross-cultural validity refers to the degree to which the performance of the items in the translated or culturally adapted instrument adequately reflects the performance of the items in the original version of the instrument [13, 31]. Cross-cultural validity is important to ensure that a measurement instrument can accurately measure what it is intended to measure among different target populations [32]. The evaluation of cross-cultural validity should be conducted across different groups, cultures, and languages [13, 31, 33]. We recommend that it be conducted for NRC instruments in different groups such as clinical nurses and nursing students, as well as across different cultures and languages to ensure their reliability across different contexts.

The study recommended all NRC instruments as Grade B. However, the lack of specific information regarding the evaluation of content and construct validity may have influenced this rating. Although all NRC instruments could be recommended for use, but further studies are necessary to confirm the reliability of all NRC instruments. It is also important to note that the stringent evaluation method of COSMIN bases the score of each measurement property on the lowest-scoring item across all items. This approach may result in lower evaluations for instruments with insufficient information [25, 34, 35]. In this study, interpretability and feasibility were not evaluated, so future research is suggested to assess these properties.

We have observed that even though the of developers these NRC instruments have limited the target population (nurses/nursing students) and provided clear definitions of NRC, there was no significant difference in their definitions of NRC for nurses and nursing students. Furthermore, minimal discrepancies exist among the NRC instruments developed for distinct populations (with the exception of attitudes towards nursing research, problem finding competence, research design competence, and paper writing competence). Therefore, further research should investigate whether a distinction in NRC between different populations (nurses and nursing students) is necessary. If different populations need to have different NRC, it becomes imperative to delineate the precise implications and extent of NRC for each distinct group. Conversely, if there is no difference, all the NRC instruments can be universally used without limiting the target population.

All the NRC instruments were recommended for use under category B. Considering that the COSMIN guidelines recommend using instruments categorized as (B), and given the current widespread used of NRC instruments, it was not recommended to develop new NRC instruments. Instead, existing NRC instruments that were recommended as category (B) could be optimized to the greatest extent possible. For example, it's worth noting that the evaluation on content validity of NRC instruments ①②③④⑥ did not encompass nurses and/or nursing students (the main reason why these NRC instruments cannot be recommended as category (A)). Therefore, it is possible to consider conducting additional interviews with nurses and/or nursing students, comparing the NRC instruments ①②③④⑥ items with the interview results to estimate which NRC instruments have very good content validity. Furthermore, the evaluation results suggest the Cronbach's alpha values of some dimensions of NRC instruments ⑤⑦⑧ are below 0.70. It's imperative to undertake large-scale studies that validate these dimensions comprehensively. If the Cronbach's alpha values for all dimensions fail to reach the desired threshold even within a large-scale study, a revision of the dimensions and entries within the existing NRC instruments should be deliberated. In a broader context, future researchers are encouraged to develop novel measurement instruments guided by the COSMIN framework. Notably, the development process should incorporate qualitative interviews with the target population, specifically focusing on gauging reliability, comprehensiveness, and understandability of content validity within these instruments. Subsequently, extensive validation of internal consistency within a sizable sample of the target population is pivotal, ensuring that instruments could be categorized as (A) merit recommendation for practical use.

By summarizing the usage of all NRC instruments, we found that nurses and nursing students were currently the main focus of research using NRC instruments, and more than 50% of the research was cross-sectional. This provides a theoretical basis for nursing researchers to understand the current situation of nurses and nursing students' NRC and develop precise intervention plans to improve their NRC. It is worth noting that although RCT and Before-after study in the same patient have been conducted, there were few studies with a large sample size and a lack of longitudinal evaluation of the effectiveness of NRC intervention by nurses and/or nursing students. In addition, almost all research was conducted in China, which may be due to the fact that the majority (87.5%) of NRC instruments were first developed by Chinese researchers. Therefore, in the future, nursing researchers from different countries should improve existing NRC instruments, select appropriate NRC instrument based on specific contexts and cultural backgrounds, and conduct cross-cultural testing to clarify the NRC competence of nursing staff from different countries and provide a theoretical basis for formulating intervention measures.

### Strengths and limitations

The study has three strengths: (1) it followed the COSMIN guideline, JBI methodology, and reported following PRISMA-ScR checklist; (2) it comprehensively searched and retrieved relevant literature from English and Chinese databases; and (3) it evaluated the methodological quality of studies and instruments according to the COSMIN guideline.

Limitations of the study include the exclusion of NRC instruments published in languages other than English and Chinese, and the possibility of missing relevant literature not included in the selected databases. In addition, the NRC instruments in this scoping review were designed for nurses and/or nursing students, not for patients. Therefore, we replaced all patients with nursers/nursing students during the evaluation. COSMIN guideline suggested that it could be used as a guidance for reviews of non-PROMs. However, COSMIN guideline did not mention how to make specific modifications to steps 5–7 (evaluate content validity, internal structure (structural validity, internal consistency, cross-cultural validity), and remaining measurement properties (reliability, measurement error, criterion validity, hypotheses testing for construct validity, and responsiveness) for non-PROMs.

## Conclusion

The study recommended NRC instrument ⑦ as the most suitable among existing instruments, but calls for further research on the measurement properties of NRC instruments, especially cross-cultural validity, measurement error, and criteria validity. Additionally, researchers should evaluate and report on the interpretability and feasibility of NRC instruments, and explore the development of more reliable and feasible instruments for different nursing populations based on a unified concept of nursing research competence.

### Implications for clinical practice

This study evaluated NRC instruments' measurement properties and provides recommendations for selecting appropriate instruments. Valid and reliable NRC instruments can accurately evaluate nurses' NRC in clinical settings and provide evidence for intervention plans to improve their competence.

### Supplementary Information

**Additional file 1: Table S1. **The characteristics of eligible NRC instruments. **Table S2. **The characteristics of study populations involved in the development and validation of eligible NRC instruments. **Table S3. **An overview of the uses of all the NRC instruments. **Table S4. **Search strategy for Pubmed.** Table S5. **Search strategy for Embase.** Table S6.** Search strategy for Scopus. **Table S7. **Search strategy for Cochrane. **Table S8. **Search strategy for CINAHL.** Table S9.** Search strategy for PsycINFO.** Table S10. **Search strategy for ERIC.** Table S11.** Search strategy for ProQuest.** Table S12.** Search strategy for Wanfang.** Table S13. **Search strategy for CNKI.

## Data Availability

Not application.
