# Supplementary material for: Instruments for measuring nursing research competence: a COSMIN-based scoping review
Source: BMC Nurs. 2023 Oct 31;22:410. doi: 10.1186/s12912-023-01572-7 (PMC10617091; doi:10.1186/s12912-023-01572-7)
Supplement: Supplementary file 1 — Additional file 1: Table S1. The characteristics of eligible NRC instruments. Table S2. The characteristics of study populations involved in the development and validation of eligible NRC instruments. Table S3. An overview of the uses of all the NRC instruments. Table S4. Search strategy for Pubmed. Table S5. Search strategy for Embase. Table S6. Search strategy for Scopus. Table S7. Search strategy for Cochrane. Table S8. Search strategy for CINAHL. Table S9. Search strategy for PsycINFO. Table S10. Search strategy for ERIC. Table S11. Search strategy for ProQuest. Table S12. Search strategy for Wanfang. Table S13. Search strategy for CNKI. [file 12912_2023_1572_MOESM1_ESM.docx]

**Table S1.** The characteristics of eligible NRC instruments

| Name of instrument/Developer | Construct(s) | Country of development (Language) | Targeted population | Mode of administration | (Sub)scale(s)/Content(s) (number of items) | Response options | Range of scores/  scoring |
| --- | --- | --- | --- | --- | --- | --- | --- |
| The Student Research Competence Instrument ①/ Arthur and Wong (Arthur & Wong, 2000). | Nursing research competence ^1^ | Hong Kong, China (Traditional Chinese language) | Nursing students | Self-report | Total scale (133 items)  Demographic variables (11 items)  Research experience (6 items)  Research orientation (19 items, one open-ended)  Attitude towards research (29 items)  Perceived research competence (13 items)  Knowledge test (20 true/false items and 35 multiple choice items) | 5-point Likert scale (59 items); open-ended (19 items); true/false questions (20 items); multiple choice questions (35 items) | 59-295 scores/ 1=disagree to 5=agree ^2^ |
| The Nursing Research Questionnaire among Nurse Clinicians ②/ Gething L, et al (Gething et al., 2001). | Nursing research competence ^2^ | Australia (English) | Nurse clinicians | Self-report | Total scale (58 items)  Exploratory stage (8 items)  Literature review stage (3 items)  Design stage (12 items)  Action stage (12 items)  Data-analysis stage (8 items)  Paper writing stage (15 items) | 5-point Likert scale | 58-290 scores/—^2^ |
| The Research Competency Scale for Nursing Students ③/ Qiu, et al (Qiu et al., 2019). | Nursing research competence ^3^ | Mainland, China  (Simplified Chinese language) | Nursing students | Self-report | Total scale (24 items) ^2^ | 5-point Likert scale | 24-120 scores/ 1=very unfamiliar, 2=know little, 3=know some, 4=familiar, 5=very familiar |
| Scientific Research Competency Scale ④/ Duru, et al (Duru & Örsal, 2021). | Nursing research competence ^4^ | Turkey  (English) | Nursing professionals at undergraduate and graduate level | Self-report | Total scale (57 items)  Technical skills (27 items)  Attitude and behaviors (19 items)  Estimation capacity (8 items)  Foreign language skills (3 items) | 5-point Likert scale | 57-285 scores/ 1=very weak, 2=weak, 3=medium, 4=good, 5=very good |
| Self-evaluated Nursing Research Capacity Questionnaire ⑤/ Liu (Liu, 2004). | Nursing research competence ^5^ | Mainland, China  (Simplified Chinese language) | Nurse staff | Self-report | Total scale (40 items)  Fundamental knowledge for research (13 items)  Statistical software operations (11 items)  Statistical knowledge (7 items)  Article writing skills (9 items) | 4-point Likert scale | 0-120 scores/ 0=very unfamiliar, 1=know some, 2=familiar, 3=very familiar |
| The Nursing Scientific Research Ability Scale ⑥/ Wu, et al (Wu et al., 2016). | Nursing research competence ^2^ | Mainland, China  (Simplified Chinese language) | Nurse staff | Self-report | Total scale (35 items)  Thesis and subject (8 items)  Scientific research award/achievement (3 items)  Research practice ability (11 items)  Ability of profession and problems finding (8 items)  Communication and collaboration ability (5 items) | 5-point Likert scale | 35-175 scores/ 0=very unfamiliar, 1=know little, 2=know some, 3=familiar, 4=very familiar |
| Self-evaluated Nursing Research Capacity Questionnaire (refined) ⑦/ Pan (Pan, 2011). | Nursing research competence ^6^ | Mainland, China  (Simplified Chinese language) | Nurse staff | Self-report | Total scale (37 items)  Article writing competency (8 items)  Data analysis competency (7 items)  Research and practice competency (6 items)  Research design competency (5 items)  Information retrieval competency (6 items)  Problem finding competency (5 items) | 5-point Likert scale | 0-148 scores/ 0=very unfamiliar, 1=know little, 2=know some, 3=familiar, 4=very familiar |
| Self-rating Research Capacity Scale of Nursing Staff ⑧  / Yin, et al (Yin et al., 2016). | Nursing research competence ^2^ | Mainland, China  (Simplified Chinese language) | Nurse staff | Self-report | Total scale (44 items)  Research design competency (6 items)  Research and practice competency (6 items)  Innovation competency (8 items)  Problem finding competency (6 items)  Information retrieval competency (7 items)  Data analysis competency (5 items)  Article writing competency (6 items) | 5-point Likert scale | 0-176 scores/ 0=very unfamiliar, 1=know little, 2=know some, 3=familiar, 4=very familiar |

Note:

^1^ The author defined NRC as research knowledge, attitudes, skills, and research orientation.

^2^ The detailed information was not found in the studies and was not available after trying to contact the corresponding author.

^3^ The author defined NRC as 1) to systematically summarize clinical experiences as well as literature to find researchable problems and formulate innovative research questions relevant to nursing, 2) to collect, analyze and explain data related to those questions, and 3) to apply knowledge accumulated in this way to solve problems innovatively.

^4^ The author defined research as ‘a process of collecting, analysing, interpreting and evaluating data in a planned and systematic way with the aim of seeking reliable solutions for problems’, and defined competency as ‘a combination of knowledge, personal attributes, interests, experiences and capabilities related to the job which enables their owners to play a role above the average level’.

^5^ The author defined NRC as 1) mastering the basic knowledge, methods, and literature retrieval skills, 2) mastered the knowledge of statistics, and 3) mastering the operating skills of computer statistical software, 4) mastering the writing skills of articles.

^6^ The author defined NRC as various psychological qualities and abilities formed in the process of people using existing knowledge and scientific methods to explore new knowledge and solve new problems.

**Table S2.** The characteristics of study populations involved in the development and validation of eligible NRC instruments

|  |  | Population | | |  | Instrument administration | | |  |  |
| --- | --- | --- | --- | --- | --- | --- | --- | --- | --- | --- |
| NRC instrument | Reference | N | Age  (Years)  % / Mean (SD, range) | Gender  % of female |  | Setting | Country | Language |  | Response rate |
| The Student Research Competence Instrument ① | 1 | 74 | < 40 (90.00%)  ≥ 40 (10.00%) | 83.00% |  | Clinical | Hong Kong, China | Traditional Chinese language |  | 90.00% |
| The Nursing Research Questionnaire among Nurse Clinicians ② | 1 | 96 | - ^1^ | - |  | Clinical | Australia | English |  | 13.40% |
| The Research Competency Scale for Nursing Students ③ | 1 | 146 | ≤ 20 (39.61%)  > 20 (60.09%) | 88.40% |  | Nursing school | China | Simplified Chinese |  | 97.90% |
| Scientific Research Competency Scale ④ | 1 |  |  |  |  |  |  |  |  |  |
| Self-evaluated Nursing Research Capacity Questionnaire ⑤ | 1 | 196 | ≤ 29 (27.00%)  30-39 (59.20%)  ≥ 40 (13.80%) | - |  | Clinical | China | Simplified Chinese |  | 97.90% |
| The Nursing Scientific Research Ability Scale ⑥ | 1 | 285 | 37.14 (±7.49) | - |  | Clinical | China | Simplified Chinese |  | 100.00% |
| Self-evaluated Nursing Research Capacity Questionnaire (refined) ⑦ | 1 | 245 | ≤ 30 (24.10%)  31-35 (27.80%)  36-40 (31.00%)  41-45 (10.60%)  ≥ 46 (6.50%) | - |  | Clinical | China | Simplified Chinese |  | 87.20% |
|  | 2 | 257 | - | - |  | Clinical | China | Simplified Chinese |  | 85.70% |
| Research Capacity Self-rating Scales of Nursing Staff ⑧ | 1 | 547 | < 40 (85.92%)  ≥ 40 (14.08%) | 100.00% |  | Clinical | China | Simplified Chinese |  | 97.8% |

Note

^1^ ‘-’ means the relevant content was not available

**Table S3.** An overview of the uses of all the NRC instruments

| The instrument of NRC used | Total number of studies using this tool | Country (n/%) | Year (n/%) | Study design (n/%) | Participants (n/%) | Sample size (n/%) |
| --- | --- | --- | --- | --- | --- | --- |
| The Nursing Research Questionnaire among Nurse Clinicians ② | 1 | Norway (1/100%) | 2012 (1/100%) | Cross-sectional study (1/100%) | Nurses (1/100%) | 364 (1/100%) |
| The Research Competency Scale for Nursing Students ③ | 2 | Kingdom of Saudi Arabia (2/100%) | 2022 (2/100%) | Cross-sectional study (2/100%) | Nursing students (2/100%) | 200-250 (2/100%) |
| Self-evaluated Nursing Research Capacity of Questionnaire ⑤ | 38 | China (38/100%) | 2009-2014 (20/52.6%)  2015-2019 (18/47.4%) | Cross-sectional study (31/81.6%)  RCT (3/7.9%)  Before-after study in the same patient (4/10.5%) | Nurses (23/60.5%)  Nursing students (6/15.8%)  Nursing teacher (1/2.6%)  The first author of "Nursing Research" journal (8/21.1%) | Cross-sectional study:  <100 (3/7.9%)  100-200 (7/22.6%)  201-300 (4/10.5%)  301-400 (3/7.9%)  >400 (14/45.1%)  RCT:  Intervention group:  10-40 (1/33.3%)  41-100 (0)  >100 (2/66.7%)  Control group:  10-40 (1/33.3%)  41-100 (0)  >100 (2/66.7%)  Before-after study in the same patient:  20-60 (3/75%)  61-100 (0)  >100 (1/25%) |
| The Nursing Scientific Research Competence Scale ⑥ | 2 | China (2/100%) | 2015-2020 (2/100%) | Cross-sectional study (2/100%) | Nurses (2/100%) | 100-200 (1/50%)  200-300 (1/50%) |
| Self-evaluated Nursing Research Capacity of Questionnaire (refined) ⑦ | 127 | China (127/100%) | 2011-2016 (42/33.1%)  2017-2022 (84/66.1%)  2023 (1/0.8%) | Cross-sectional study (91/71.7%)  RCT (11/8.7%)  Before-after study in the same patient (23/18.1%)  A quasi-experimental study (1/0.8%)  Review (1/0.8%) | Nurses (77/60.6%)  Nursing students (47/37.0%)  The first author of "Nursing Research" journal (3/2.4%) | Cross-sectional study:  <100 (4/4.4%)  100-200 (29/31.9%)  201-300 (19/20.9%%)  301-400 (5/5.5%)  >400 (34/37.3%%)  RCT:  Intervention group:  10-40 (2/18.2%)  41-100 (7/63.6%)  >100 (2/18.2%)  Control group:  10-50 (2/18.2%)  51-100 (7/63.6%)  >100 (2/18.2%)  Before-after study in the same patient:  20-60 (7/30.4%)  61-100 (8/34.8%)  >100 (8/34.8%)  A quasi-experimental study:  108 (1/100%) |
| Research Capacity Self-Rating Scales of Nursing Staff ⑧ | 7 | China (7/100%) | 2019-2023 (7/100%) | Cross-sectional study (3/42.9%)  RCT (1/14.2%)  Before-after study in the same patient (3/42.9%) | Nurses (5/71.4%)  Nursing students (2/28.6%) | Cross-sectional study:  >500 (3/100%)  RCT:  Intervention group:  68 (1/100%)  Control group: |
| Self-designed scale | 23 | China (23/100%) | 2010-2015 (12/52.2%)  2016-2021 (11/47.8%) | Cross-sectional study (17/73.8%)  RCT (3/13.1%)  Before-after study in the same patient (3/13.1%) | Nurses (13/56.5%)  Nursing students (9/39.1%)  The first author of "Nursing Research" journal (1/4.4%) | 68 (1/100%)  Before-after study in the same patient:  <100 (3/100%)  Cross-sectional study:  <100 (3/17.6%)  101-200 (7/41.2%)  201-300 (2/11.8%)  >300 (5/29.4%)  RCT:  Intervention group:  10-50 (0)  51-100 (2/66.7%)  >100 (1/33.3%)  Control group:  10-50 (1/33.3%)  51-100 (2/66.7%)  >100 (0)  Before-after study in the same patient:  <100 (3/100%) |

**Table S4.** Search strategy for Pubmed

| 1 | #1 | Search: nurs* [Title] |
| --- | --- | --- |
| 2 | #2 | Search: research[Title] |
| 3 | #3 | Search: professional competenc*[MeSH Terms] OR ((competenc*[Title/Abstract] OR capabilit^*^[Title/Abstract] OR capacit*[Title/Abstract] OR abilit*[Title/Abstract]) OR (knowledge[Title/Abstract] AND skill*[Title/Abstract])) |
| 4 | #4 | Search: assess*[Title/Abstract] OR measur*[Title/Abstract] OR evaluat*[Title/Abstract] OR collec*[Title/Abstract] OR survey*[Title/Abstract] OR instrument*[Title/Abstract] OR scale*[Title/Abstract] OR questionnaire*[Title/Abstract] OR tool*[Title/Abstract] |
| 5 | #5 | Search: (instrumentation[sh] OR methods[sh] OR “Validation Study”[pt] OR “Comparative Study”[pt] OR “psychometrics”[MeSH] OR psychometr*[tiab] OR clinimetr*[tw] OR clinometr*[tw] OR “outcome assessment, health care”[MeSH] OR “outcome assessment”[tiab] OR “outcome measure*”[tw] OR “observer variation”[MeSH] OR “observer variation”[tiab] OR “Health Status Indicators”[Mesh] OR “reproducibility of results”[MeSH] OR reproducib*[tiab] OR “discriminant analysis”[MeSH] OR reliab*[tiab] OR unreliab*[tiab] OR valid*[tiab] OR “coefficient of variation”[tiab] OR coefficient[tiab] OR homogeneity[tiab] OR homogeneous[tiab] OR “internal consistency”[tiab] OR (cronbach*[tiab] AND (alpha[tiab] OR alphas[tiab])) OR (item[tiab] AND (correlation*[tiab] OR selection*[tiab] OR reduction*[tiab])) OR agreement[tw] OR precision[tw] OR imprecision[tw] OR “precise values”[tw] OR test-retest[tiab] OR (test[tiab] AND retest[tiab]) OR (reliab*[tiab] AND (test[tiab] OR retest[tiab])) OR stcompetence[tiab] OR interrater[tiab] OR inter-rater[tiab] OR intrarater[tiab] OR intra-rater[tiab] OR intertester[tiab] OR inter-tester[tiab] OR intratester[tiab] OR intra-tester[tiab] OR interobserver[tiab] OR inter-observer[tiab] OR intraobserver[tiab] OR intra-observer[tiab] OR intertechnician[tiab] OR inter-technician[tiab] OR intratechnician[tiab] OR intra-technician[tiab] OR interexaminer[tiab] OR inter-examiner[tiab] OR intraexaminer[tiab] OR intra-examiner[tiab] OR interassay[tiab] OR inter-assay[tiab] OR intraassay[tiab] OR intra-assay[tiab] OR interindividual[tiab] OR inter-individual[tiab] OR intraindividual[tiab] OR intra-individual[tiab] OR interparticipant[tiab] OR inter-participant[tiab] OR intraparticipant[tiab] OR intra-participant[tiab] OR kappa[tiab] OR kappa’s[tiab] OR kappas[tiab] OR repeatab*[tw] OR ((replicab*[tw] OR repeated[tw]) AND (measure[tw] OR measures[tw] OR findings[tw] OR result[tw] OR results[tw] OR test[tw] OR tests[tw])) OR generaliza*[tiab] OR generalisa*[tiab] OR concordance[tiab] OR (intraclass[tiab] AND correlation*[tiab]) OR discriminative[tiab] OR “known group”[tiab] OR “factor analysis”[tiab] OR “factor analyses”[tiab] OR “factor structure”[tiab] OR “factor structures”[tiab] OR dimension*[tiab] OR subscale*[tiab] OR (multitrait[tiab] AND scaling[tiab] AND (analysis[tiab] OR analyses[tiab])) OR “item discriminant”[tiab] OR “interscale correlation*”[tiab] OR error[tiab] OR errors[tiab] OR “individual varicompetence”[tiab] OR “interval varicompetence”[tiab] OR “rate varicompetence”[tiab] OR (varicompetence[tiab] AND (analysis[tiab] OR values[tiab])) OR (uncertainty[tiab] AND (measurement[tiab] OR measuring[tiab])) OR “standard error of measurement”[tiab] OR sensitiv*[tiab] OR responsive*[tiab] OR (limit[tiab] AND detection[tiab]) OR “minimal detectable concentration”[tiab] OR interpretab*[tiab] OR ((minimal[tiab] OR minimally[tiab] OR clinical[tiab] OR clinically[tiab]) AND (important[tiab] OR significant[tiab] OR detectable[tiab]) AND (change[tiab] OR difference[tiab])) OR (small*[tiab] AND (real[tiab] OR detectable[tiab]) AND (change[tiab] OR difference[tiab])) OR “meaningful change”[tiab] OR “ceiling effect”[tiab] OR “floor effect”[tiab] OR “Item response model”[tiab] OR IRT[tiab] OR Rasch[tiab] OR “Differential item functioning”[tiab] OR DIF[tiab] OR “computer adaptive testing”[tiab] OR “item bank”[tiab] OR “cross-cultural equivalence”[tiab]) |
| 6 | #6 | (“Address”[Publication Type] OR “biography”[Publication Type] OR “case reports”[Publication Type] OR “comment”[Publication Type] OR “directory”[Publication Type] OR “editorial”[Publication Type] OR “festschrift”[Publication Type] OR “interview”[Publication Type] OR “Lecture”[Publication Type] OR “legal case”[Publication Type] OR “legislation”[Publication Type] OR “letter”[Publication Type] OR “news”[Publication Type] OR “newspaper article”[Publication Type] OR “patient education handout”[Publication Type] OR “popular work”[Publication Type] OR “congress”[Publication Type] OR “consensus development conference”[Publication Type] OR “consensus development conference, nih”[Publication Type] OR “practice guideline”[Publication Type]) NOT (“animals”[MeSH Terms] NOT “humans”[MeSH Terms]) |
| 7 | #7 | (#1 AND #2 AND #3 AND #4 AND #5) NOT #6 |

**Table S5.** Search strategy for Embase

| 1 | #1 | Search: 'nurs*':ti |
| --- | --- | --- |
| 2 | #2 | Search: 'research':ti |
| 3 | #3 | Search: 'professional competence'/exp OR (competenc*:ab,ti OR 'competence'/exp OR 'competency'/exp OR 'competencies'/exp OR 'capabilit*':ab,ti OR 'capacitance'/exp OR 'capacity'/exp OR 'abilit*':ab,ti OR ('knowledge'/exp AND 'skill'/exp OR 'skill':ab,ti)) |
| 4 | #4 | Search: 'assess*':ab,ti OR 'measurement'/exp OR 'measur*':ab,ti OR 'evaluat*':ab,ti OR 'collec*':ab,ti OR 'survey'/exp OR 'surveys'/exp OR 'survey*':ab,ti OR 'instrument*':ab,ti OR 'scale'/exp OR 'scale*':ab,ti OR 'questionnaire'/exp OR 'questionnaire*':ab,ti OR 'tool'/exp OR 'tool*':ab,ti |
| 5 | #5 | Search: 'intermethod comparison'/exp OR 'data collection method'/exp OR 'validation study'/exp OR 'feasibility study'/exp OR 'pilot study'/exp OR 'psychometry'/exp OR 'reproducibility'/exp OR reproducib*:ab,ti OR 'audit':ab,ti OR psychometr*:ab,ti OR clinimetr*:ab,ti OR clinometr*:ab,ti OR 'observer variation'/exp OR 'observer variation':ab,ti OR 'discriminant analysis'/exp OR 'validity'/exp OR reliab*:ab,ti OR valid*:ab,ti OR 'coefficient':ab,ti OR 'internal consistency':ab,ti OR (cronbach*:ab,ti AND ('alpha':ab,ti OR 'alphas':ab,ti)) OR 'item correlation':ab,ti OR 'item correlations':ab,ti OR 'item selection':ab,ti OR 'item selections':ab,ti OR 'item reduction':ab,ti OR 'item reductions':ab,ti OR 'agreement':ab,ti OR 'precision':ab,ti OR 'imprecision':ab,ti OR 'precise values':ab,ti OR 'test- retest':ab,ti OR ('test':ab,ti AND 'retest':ab,ti) OR (reliab*:ab,ti AND ('test':ab,ti OR 'retest':ab,ti)) OR 'stcompetence':ab,ti OR 'interrater':ab,ti OR 'inter-rater':ab,ti OR 'intrarater':ab,ti OR 'intra-rater':ab,ti OR 'intertester':ab,ti OR 'inter- tester':ab,ti OR 'intratester':ab,ti OR 'intratester':ab,ti OR 'interobeserver':ab,ti OR 'inter-observer':ab,ti OR 'intraobserver':ab,ti OR 'intraobserver':ab,ti OR 'intertechnician':ab,ti OR 'inter-technician':ab,ti OR 'intratechnician':ab,ti OR 'intratechnician':ab,ti OR 'interexaminer':ab,ti OR 'inter-examiner':ab,ti OR 'intraexaminer':ab,ti OR 'intraexaminer':ab,ti OR 'interassay':ab,ti OR 'inter- assay':ab,ti OR 'intraassay':ab,ti OR 'intra-assay':ab,ti OR 'interindividual':ab,ti OR 'inter-individual':ab,ti OR 'intraindividual':ab,ti OR 'intra-individual':ab,ti OR 'interparticipant':ab,ti OR 'inter-participant':ab,ti OR 'intraparticipant':ab,ti OR 'intraparticipant':ab,ti OR 'kappa':ab,ti OR 'kappas':ab,ti OR 'coefficient of variation':ab,ti OR repeatab*:ab,ti OR (replicab*:ab,ti OR 'repeated':ab,ti AND ('measure':ab,ti OR 'measures':ab,ti OR 'findings':ab,ti OR 'result':ab,ti OR 'results':ab,ti OR 'test':ab,ti OR 'tests':ab,ti)) OR generaliza*:ab,ti OR generalisa*:ab,ti OR 'concordance':ab,ti OR ('intraclass':ab,ti AND correlation*:ab,ti) OR 'discriminative':ab,ti OR 'known group':ab,ti OR 'factor analysis':ab,ti OR 'factor analyses':ab,ti OR 'factor structure':ab,ti OR 'factor structures':ab,ti OR 'dimensionality':ab,ti OR subscale*:ab,ti OR 'multitrait scaling analysis':ab,ti OR 'multitrait scaling analyses':ab,ti OR 'item discriminant':ab,ti OR 'interscale correlation':ab,ti OR 'interscale correlations':ab,ti OR ('error':ab,ti OR 'errors':ab,ti AND (measure*:ab,ti OR correlat*:ab,ti OR evaluat*:ab,ti OR 'accuracy':ab,ti OR 'accurate':ab,ti OR 'precision':ab,ti OR 'mean':ab,ti)) OR 'individual varicompetence':ab,ti OR 'interval varicompetence':ab,ti OR 'rate varicompetence':ab,ti OR 'varicompetence analysis':ab,ti OR ('uncertainty':ab,ti AND ('measurement':ab,ti OR 'measuring':ab,ti)) OR 'standard error of measurement':ab,ti OR sensitiv*:ab,ti OR responsive*:ab,ti OR ('limit':ab,ti AND 'detection':ab,ti) OR 'minimal detectable concentration':ab,ti OR interpretab*:ab,ti OR (small*:ab,ti AND ('real':ab,ti OR 'detectable':ab,ti) AND ('change':ab,ti OR 'difference':ab,ti)) OR 'meaningful change':ab,ti OR 'minimal important change':ab,ti OR 'minimal important difference':ab,ti OR 'minimally important change':ab,ti OR 'minimally important difference':ab,ti OR 'minimal detectable change':ab,ti OR 'minimal detectable difference':ab,ti OR 'minimally detectable change':ab,ti OR 'minimally detectable difference':ab,ti OR 'minimal real change':ab,ti OR 'minimal real difference':ab,ti OR 'minimally real change':ab,ti OR 'minimally real difference':ab,ti OR 'ceiling effect':ab,ti OR 'floor effect':ab,ti OR 'item response model':ab,ti OR 'irt':ab,ti OR 'rasch':ab,ti OR 'differential item functioning':ab,ti OR 'dif':ab,ti OR 'computer adaptive testing':ab,ti OR 'item bank':ab,ti OR 'cross-cultural equivalence':ab,ti |
| 6 | #6 | Search: ('address':it OR 'biography':it OR 'case reports':it OR 'comment':it OR 'directory':it OR 'editorial':it OR 'festschrift':it OR 'interview':it OR 'lectures':it OR 'legal cases':it OR 'legislation':it OR 'letter':it OR 'news':it OR 'newspaper article':it OR 'patient education handout':it OR 'popular works':it OR 'congresses':it OR 'consensus development conference':it OR 'consensus development conference':it OR 'practice guideline':it) NOT ('animal'/exp NOT 'human'/exp) |
| 7 | #7 | #1 AND #2 AND #3 AND #4 AND #5 NOT #6 |

**Table S6.** Search strategy for Scopus

| 1 | #1 | Search: TITLE ( nurs* ) |
| --- | --- | --- |
| 2 | #2 | Search: TITLE (research) |
| 3 | #3 | Search: TITLE-ABS-KEY ( "professional competenc*" ) OR ((TITLE-ABS-KEY ( "competenc*" ) OR TITLE-ABS-KEY ( "capabilit*" ) OR TITLE-ABS-KEY ( "capacit*" ) OR TITLE-ABS-KEY ( "competence*" )) OR (TITLE-ABS-KEY ( "knowledge" ) AND TITLE-ABS-KEY ( "skill*" ))) |
| 4 | #4 | Search: TITLE-ABS-KEY ( "assess*" ) OR TITLE-ABS-KEY ( "measur*" ) OR TITLE-ABS-KEY ( "evaluat*" ) OR TITLE-ABS-KEY ( "collec*" ) OR TITLE-ABS-KEY ( "survey*" ) OR TITLE-ABS-KEY ( "instrument*" ) OR TITLE-ABS-KEY ( "scale*" ) OR TITLE-ABS-KEY ( " questionnaire*" ) OR TITLE-ABS-KEY ( " tool*" ) |
| 5 | #5 | Search: ( TITLE-ABS-KEY ( "instrumentation" )  OR  TITLE-ABS-KEY ( "methods" )  OR  TITLE-ABS-KEY ( "psychometr*" )  OR  ALL ( clinimetr* )  OR  ALL ( clinometr* )  OR  TITLE-ABS-KEY ( "outcome assessment, health care" )  OR  TITLE-ABS-KEY ( "outcome assessment" )  OR  TITLE-ABS-KEY ( "outcome measure*" )  OR  TITLE-ABS-KEY ( "observer variation" )  OR  TITLE-ABS-KEY ( "Health Status Indicators" )  OR  TITLE-ABS-KEY ( "reproducibility of results" )  OR  TITLE-ABS-KEY ( "reproducib*" )  OR  TITLE-ABS-KEY ( "discriminant analysis" )  OR  TITLE-ABS-KEY ( "reliab*" )  OR  TITLE-ABS-KEY ( "unreliab*" )  OR  TITLE-ABS-KEY ( "valid*" )  OR  TITLE-ABS-KEY ( "coefficient of variation" )  OR  TITLE-ABS-KEY ( "coefficient" )  OR  TITLE-ABS-KEY ( "homogeneity" )  OR  TITLE-ABS-KEY ( "homogeneous" )  OR  TITLE-ABS-KEY ( "internal consistency" )  OR  ( TITLE-ABS-KEY ( "cronbach*" )  AND  ( TITLE-ABS-KEY ( "alpha" )  OR  TITLE-ABS-KEY ( "alphas" ) ) )  OR  ( TITLE-ABS-KEY ( "item" )  AND  ( TITLE-ABS-KEY ( correlation* )  OR  TITLE-ABS-KEY ( "selection*" )  OR  TITLE-ABS-KEY ( "reduction*" ) ) )  OR  ALL ( agreement )  OR  ALL ( precision )  OR  ALL ( imprecision )  OR  ALL ( "precise values" )  OR  TITLE-ABS-KEY ( "test-retest" )  OR  ( TITLE-ABS-KEY ( "test" )  AND  TITLE-ABS-KEY ( "retest" ) )  OR  ( TITLE-ABS-KEY ( "reliab*" )  AND  ( TITLE-ABS-KEY ( "test" )  OR  TITLE-ABS-KEY ( "retest" ) ) )  OR  TITLE-ABS-KEY ( "stcompetence" )  OR  TITLE-ABS-KEY ( "interrater" )  OR  TITLE-ABS-KEY ( "inter-rater" )  OR  TITLE-ABS-KEY ( "intrarater" )  OR  TITLE-ABS-KEY ( "intra-rater" )  OR  TITLE-ABS-KEY ( "intertester" )  OR  TITLE-ABS-KEY ( "inter-tester" )  OR  TITLE-ABS-KEY ( "intratester" )  OR  TITLE-ABS-KEY ( "intra-tester" )  OR  TITLE-ABS-KEY ( "interobserver" )  OR  TITLE-ABS-KEY ( "inter-observer" )  OR  TITLE-ABS-KEY ( "intraobserver" )  OR  TITLE-ABS-KEY ( "intra-observer" )  OR  TITLE-ABS-KEY ( "intertechnician" )  OR  TITLE-ABS-KEY ( "inter-technician" )  OR  TITLE-ABS-KEY ( "intratechnician" )  OR  TITLE-ABS-KEY ( "intra-technician" )  OR  TITLE-ABS-KEY ( "interexaminer" )  OR  TITLE-ABS-KEY ( "inter-examiner" )  OR  TITLE-ABS-KEY ( " intraexaminer" )  OR  TITLE-ABS-KEY ( "intra-examiner" )  OR  TITLE-ABS-KEY ( "interassay" )  OR  TITLE-ABS-KEY ( "inter-assay" )  OR  TITLE-ABS-KEY ( "intraassay" )  OR  TITLE-ABS-KEY ( "intra-assay" )  OR  TITLE-ABS-KEY ( "interindividual" )  OR  TITLE-ABS-KEY ( "inter-individual" )  OR  TITLE-ABS-KEY ( "intraindividual" )  OR  TITLE-ABS-KEY ( "intra-individual" )  OR  TITLE-ABS-KEY ( "interparticipant" )  OR  TITLE-ABS-KEY ( "inter-participant" )  OR  TITLE-ABS-KEY ( "intraparticipant" )  OR  TITLE-ABS-KEY ( "intra-participant" )  OR  TITLE-ABS-KEY ( "kappa" )  OR  TITLE-ABS-KEY ( "kappa's" )  OR  TITLE-ABS-KEY ( "kappas" )  OR  ALL ( "repeatab*" )  OR  ( ( ALL ( "replicab*" )  OR  ALL ( "repeated" ) )  AND  ( ALL ( "measure" )  OR  ALL ( "measures" )  OR  ALL ( "findings" )  OR  ALL ( "result" )  OR  ALL ( "results" )  OR  ALL ( "test" )  OR  ALL ( "tests" ) ) )  OR  TITLE-ABS-KEY ( "generaliza*" )  OR  TITLE-ABS-KEY ( "generalisa*" )  OR  TITLE-ABS-KEY ( "generalisa*" )  OR  TITLE-ABS-KEY ( "concordance" )  OR  ( TITLE-ABS-KEY ( "intraclass" )  AND  TITLE-ABS-KEY ( "correlation*" ) )  OR  TITLE-ABS-KEY ( " discriminative" )  OR  TITLE-ABS-KEY ( "known group" )  OR  TITLE-ABS-KEY ( "factor analysis" )  OR  TITLE-ABS-KEY ( "factor analyses" )  OR  TITLE-ABS-KEY ( "factor structure" )  OR  TITLE-ABS-KEY ( "factor structures" )  OR  TITLE-ABS-KEY ( "dimension*" )  OR  TITLE-ABS-KEY ( "subscale*" )  OR  ( TITLE-ABS-KEY ( "multitrait" )  AND  TITLE-ABS-KEY ( "scaling" )  AND  ( TITLE-ABS-KEY ( "analysis" )  OR  TITLE-ABS-KEY ( "analyses" ) ) )  OR  TITLE-ABS-KEY ( "item discriminant" )  OR  TITLE-ABS-KEY ( "interscale correlation*" )  OR  TITLE-ABS-KEY ( "error" )  OR  TITLE-ABS-KEY ( "errors" )  OR  TITLE-ABS-KEY ( "individual varicompetence" )  OR  TITLE-ABS-KEY ( "interval varicompetence" )  OR  TITLE-ABS-KEY ( "rate varicompetence" )  OR  ( TITLE-ABS-KEY ( "varicompetence" )  AND  ( TITLE-ABS-KEY ( "analysis" )  OR  TITLE-ABS-KEY ( "values" ) ) )  OR  ( TITLE-ABS-KEY ( "uncertainty" )  AND  ( TITLE-ABS-KEY ( "measurement" )  OR  TITLE-ABS-KEY ( "measuring" ) ) )  OR  TITLE-ABS-KEY ( "standard error of measurement" )  OR  TITLE-ABS-KEY ( "sensitiv*" )  OR  TITLE-ABS-KEY ( "responsive*" )  OR  ( TITLE-ABS-KEY ( "limit" )  AND  TITLE-ABS-KEY ( "detection" ) )  OR  TITLE-ABS-KEY ( "minimal detectable concentration" )  OR  TITLE-ABS-KEY ( "interpretab*" )  OR  ( ( TITLE-ABS-KEY ( "minimal" )  OR  TITLE-ABS-KEY ( "minimally" )  OR  TITLE-ABS-KEY ( "clinical" )  OR  TITLE-ABS-KEY ( "clinically" ) )  AND  ( TITLE-ABS-KEY ( "important" )  OR  TITLE-ABS-KEY ( "significant" )  OR  TITLE-ABS-KEY ( "detectable" ) )  AND  ( TITLE-ABS-KEY ( "change" )  OR  TITLE-ABS-KEY ( "difference" ) ) )  OR  ( TITLE-ABS-KEY ( "small*" )  AND  ( TITLE-ABS-KEY ( "real" )  OR  TITLE-ABS-KEY ( "detectable" ) )  AND  ( TITLE-ABS-KEY ( "change" )  OR  TITLE-ABS-KEY ( "difference" ) ) )  OR  TITLE-ABS-KEY ( "meaningful change" )  OR  TITLE-ABS-KEY ( "ceiling effect" )  OR  TITLE-ABS-KEY ( "floor effect" )  OR  TITLE-ABS-KEY ( "Item response model" )  OR  TITLE-ABS-KEY ( "IRT" )  OR  TITLE-ABS-KEY ( "Rasch" )  OR  TITLE-ABS-KEY ( "Differential item functioning" )  OR  TITLE-ABS-KEY ( "DIF" )  OR  TITLE-ABS-KEY ( "computer adaptive testing" )  OR  TITLE-ABS-KEY ( "item bank" )  OR  TITLE-ABS-KEY ( "cross-cultural equivalence" ) ) |
| 6 | #6 | #1 AND #2 AND #3 AND #4 AND #5 |

**Table S7.** Search strategy for Cochrane

| 1 | #1 | Search: (nurs*):ti |
| --- | --- | --- |
| 2 | #2 | Search: (research):ti |
| 3 | #3 | Search: MeSH descriptor: [Professional Competence] explode all trees OR ((professional competenc*):ti,ab,kw OR (competenc*):ti,ab,kw OR (capabilit^*^):ti,ab,kw OR (capacit*):ti,ab,kw OR (abilit*):ti,ab,kw OR (MeSH descriptor: [Knowledge] explode all trees AND (skill*):ti,ab,kw)) |
| 4 | #4 | Search: (assess*):ti,ab,kw OR (measur*):ti,ab,kw OR (evaluat*):ti,ab,kw OR (collec*):ti,ab,kw OR (survey*):ti,ab,kw OR (instrument*):ti,ab,kw OR (scale*):ti,ab,kw OR (questionnaire*):ti,ab,kw OR (tool*):ti,ab,kw |
| 5 | #5 | Search: ( (instrumentation):ti,ab,kw OR MeSH descriptor: [Methods] explode all trees OR (Validation Study):pt OR (Comparative Study):pt OR MeSH descriptor: [Psychometrics] explode all trees OR (psychometr*):ti,ab,kw OR (clinimetr*) OR (clinometr*) OR MeSH descriptor: [Outcome Assessment, Health Care] explode all trees OR (outcome assessment):ti,ab,kw OR (outcome measure*) OR MeSH descriptor: [Observer Variation] explode all trees OR (observer variation):ti,ab,kw OR MeSH descriptor: [Health Status Indicators] explode all trees OR MeSH descriptor: [Reproducibility of Results] explode all trees OR (reproducib*):ti,ab,kw OR MeSH descriptor: [Discriminant Analysis] explode all trees OR (reliab*):ti,ab,kw OR (unreliab*):ti,ab,kw OR (valid*):ti,ab,kw OR (coefficient of variation):ti,ab,kw OR (coefficient):ti,ab,kw OR (homogeneity):ti,ab,kw OR (homogeneous):ti,ab,kw OR (internal consistency):ti,ab,kw OR (cronbach*):ti,ab,kw AND ((alpha):ti,ab,kw OR (alphas):ti,ab,kw)) OR ((item):ti,ab,kw AND (correlation*):ti,ab,kw OR (selection*):ti,ab,kw OR (reduction*):ti,ab,kw) OR (agreement) OR (precision) OR (imprecision) OR (“precise values”) OR (test-retest):ti,ab,kw OR ((test):ti,ab,kw AND (retest):ti,ab,kw) OR ((reliab*):ti,ab,kw AND ((test t):ti,ab,kw OR (retest):ti,ab,kw)) OR (stcompetence):ti,ab,kw OR (interrater):ti,ab,kw OR (inter-rater):ti,ab,kw OR (intrarater):ti,ab,kw OR (intra-rater):ti,ab,kw OR (intertester):ti,ab,kw OR (inter-tester):ti,ab,kw OR (intratester):ti,ab,kw OR (intra-tester):ti,ab,kw OR (interobserver):ti,ab,kw OR (inter-observer):ti,ab,kw OR (intraobserver):ti,ab,kw OR (intra-observer):ti,ab,kw OR (intertechnician):ti,ab,kw OR (inter-technician):ti,ab,kw OR (intratechnician):ti,ab,kw OR (intra-technician):ti,ab,kw OR (interexaminer):ti,ab,kw OR (inter-examiner):ti,ab,kw OR (intraexaminer):ti,ab,kw OR (intra-examiner):ti,ab,kw OR (interassay):ti,ab,kw OR (inter-assay):ti,ab,kw OR (intraassay):ti,ab,kw OR (intra-assay):ti,ab,kw OR (interindividual):ti,ab,kw OR (inter-individual):ti,ab,kw OR (intraindividual):ti,ab,kw OR (intra-individual):ti,ab,kw OR (interparticipant):ti,ab,kw OR (inter-participant):ti,ab,kw OR (intraparticipant):ti,ab,kw OR (intra-participant):ti,ab,kw OR (kappa):ti,ab,kw OR (kappa’s):ti,ab,kw OR (kappas):ti,ab,kw OR (repeatab*) OR (((replicab*) OR (repeated)) AND ((measure) OR (measures) OR (findings) OR (result) OR (results) OR (test) OR (tests))) OR (generaliza*):ti,ab,kw OR (generalisa*):ti,ab,kw OR (concordance):ti,ab,kw OR ((intraclass):ti,ab,kw AND (correlation*):ti,ab,kw) OR (discriminative):ti,ab,kw OR (“known group”):ti,ab,kw OR (“factor analysis”):ti,ab,kw OR (“factor analyses”):ti,ab,kw OR (“factor structure”):ti,ab,kw OR (“factor structures”):ti,ab,kw OR (dimension*):ti,ab,kw OR (subscale*):ti,ab,kw OR ((multitrait):ti,ab,kw AND (scaling):ti,ab,kw AND ((analysis):ti,ab,kw OR (analyses):ti,ab,kw)) OR (“item discriminant”):ti,ab,kw OR (interscale correlation*):ti,ab,kw OR (error):ti,ab,kw OR (errors):ti,ab,kw OR (“individual varicompetence”):ti,ab,kw OR (“interval varicompetence”):ti,ab,kw OR (“rate varicompetence”):ti,ab,kw OR ((varicompetence):ti,ab,kw AND (analysis):ti,ab,kw OR (values):ti,ab,kw) OR (MeSH descriptor: [Uncertainty] explode all trees AND ((measurement):ti,ab,kw OR (measuring):ti,ab,kw)) OR (“standard error of measurement”):ti,ab,kw OR (sensitiv*):ti,ab,kw OR (responsive*):ti,ab,kw OR ((limit):ti,ab,kw AND (detection):ti,ab,kw) OR (“minimal detectable concentration”):ti,ab,kw OR (interpretab*):ti,ab,kw OR (((minimal):ti,ab,kw OR (minimally) :ti,ab,kw OR (clinical) :ti,ab,kw OR (clinically) :ti,ab,kw) AND ((important) :ti,ab,kw OR (significant) :ti,ab,kw OR (detectable) :ti,ab,kw) AND ((change):ti,ab,kw OR (difference):ti,ab,kw)) OR ((small*):ti,ab,kw AND ((real):ti,ab,kw OR (detectable):ti,ab,kw) AND ((change):ti,ab,kw OR (difference):ti,ab,kw)) OR (“meaningful change”):ti,ab,kw OR (“ceiling effect”):ti,ab,kw OR (“floor effect”):ti,ab,kw OR (“Item response model”):ti,ab,kw OR (IRT):ti,ab,kw OR (Rasch):ti,ab,kw OR (“Differential item functioning”):ti,ab,kw OR (DIF):ti,ab,kw OR (“computer adaptive testing”):ti,ab,kw OR (“item bank”):ti,ab,kw OR (“cross-cultural equivalence”):ti,ab,kw) |
| 6 | #6 | ((“Address”):pt OR (“biography”):pt OR (“case reports”):pt OR (“comment”):pt OR (“directory”):pt OR (“editorial”):pt OR (“festschrift”):pt OR (“interview”):pt OR (“Lecture”):pt OR (“legal case”):Pt OR (“legislation”):pt OR (“letter”):pt OR (“news”):pt OR (“newspaper article”):pt OR (“patient education handout”):pt OR (“popular work”):pt OR (“congress”):pt OR (“consensus development conference”):pt OR (“consensus development conference, nih”):pt OR (“practice guideline”):pt) NOT ((animals):ti,ab,kw NOT (humans):ti,ab,kw) |
| 7 | #7 | #1 AND #2 AND #3 AND #4 AND #5 NOT #6 |

**Table S8.** Search strategy for CINAHL

| 1 | #1 | Search: nurs* [Title] |
| --- | --- | --- |
| 2 | #2 | Search: research[Title] |
| 3 | #3 | Search: (MH "Professional Competence+") or (TI professional competenc* or AB professional competenc*) or ((TI competenc* or AB competenc*) or (TI capabilit* or AB capabilit*) or (TI capacit* or AB capacit*) or (TI abilit* or AB abilit*))or ((MH "Knowledge+") or (TI Knowledge or AB Knowledge) and (TI skill* or AB skill*)) |
| 4 | #4 | Search: (TI assess* or AB assess*) or (TI measur* or AB measur*) or (MH "Evaluation+") or (TI evaluat* or AB evaluat*) or (TI collec* or AB collec*) or (MH "Surveys+") or (TI survey* or AB survey*) or (TI instrument* or AB instrument*) or (MH "Scales") or (TI scale* or AB scale*) or (MH "Questionnaires+") or (TI questionnaire* or AB questionnaire*) or (TI tool* or AB tool*) |
| 5 | #5 | Search: (MH “Psychometrics”) or ( TI psychometr* or AB psychometr* ) or ( TI clinimetr* or AB clinimetr* ) or ( TI clinometr* OR AB clinometr* ) or (MH “Outcome Assessment”) or ( TI outcome assessment or AB outcome assessment ) or ( TI outcome measure* or AB outcome measure* ) or (MH “Health Status Indicators”) or (MH “Reproducibility of Results”) or (MH “Discriminant Analysis”) or ( ( TI reproducib* or AB reproducib* ) or ( TI reliab* or AB reliab* ) or ( TI unreliab* or AB unreliab* ) ) or ( ( TI valid* or AB valid* ) or ( TI coefficient or AB coefficient ) or ( TI homogeneity or AB homogeneity ) ) or ( TI homogeneous or AB homogeneous ) or ( TI “coefficient of variation” or AB “coefficient of variation” ) or ( TI “internal consistency” or AB “internal consistency” ) or (MH “Internal Consistency+”) or (MH “Relicompetence+”) or (MH “Measurement Error+”) or (MH “Content Validity+”) or “hypothesis testing” or “structural validity” or “cross-cultural validity” or (MH “Criterion-Related Validity+”) or “responsiveness” or “interpretcompetence” or ( TI reliab* or AB reliab* ) and ( (TI test or AB test) OR (TI retest or AB retest) ) or ( TI stcompetence or AB stcompetence ) or ( TI interrater or AB interrater ) or ( TI inter-rater or AB inter-rater ) or ( TI intrarater or AB intrarater ) or ( TI intra-rater or AB intrarater) or ( TI intertester or AB intertester) or (TI inter-tester or AB inter-tester) or ( TI intratester or AB intratester) or ( TI intra-tester or AB intra-tester) or ( TI interobserver or AB interobserver) or (TI inter-observer or AB inter-observer ) or ( TI intraobserver or AB intraobserver) or ( TI intra-observer or AB intra-observer) or ( TI intertechnician or AB intertechnician) or (TI inter-technician or AB inter-technician) or ( TI intratechnician or AB intratechnician ) or ( TI intra-technician or AB intra-technician ) or ( TI interexaminer or AB interexaminer ) or (TI inter-examiner or AB inter-examiner) or (TI intraexaminer or AB intraexaminer ) OR (TI intra-examiner or AB intra-examiner ) or (TI intra-examiner or AB intraexaminer) or (TI interassay or AB interassay ) or ( TI inter-assay or AB inter-assay ) or ( TI intraassay or AB intraassay) or ( TI intra-assay or AB intra-assay ) or (TI interindividual or AB interindividual) or (TI inter-individual or AB inter-individual) OR (TI intraindividual or AB intraindividual) or (TI intra-individual or AB intra-individual) or (TI interparticipant or AB interparticipant) or (TI inter-participant or AB inter-participant ) or (TI intraparticipant or AB intraparticipant) or (TI intra-participant or AB intra-participant ) or (TI kappa or AB kappa) or (TI kappa’s or AB kappa’s ) or (TI kappas or AB kappas) or (TI repeatab* or AB repeatab*) or ( TI responsive* or AB responsive* ) or ( TI interpretab* or AB interpretab* ) |
| 6 | #6 | (address OR biography OR “case reports” OR comment OR directory OR editorial OR festschrift OR interview OR lecture OR “legal case” OR legislation OR letter OR news OR “newspaper article” OR “patient education handout” OR “popular work” OR congress OR “consensus development conference” OR “consensus development conference, NIH” OR “practice guideline”).mp |
| 7 | #7 | #1 AND #2 AND #3 AND #4 AND #5 NOT #6 |

**Table S9.** Search strategy for PsycINFO

| 1 | #1 | Search: TI nurs* |
| --- | --- | --- |
| 2 | #2 | Search: TI research |
| 3 | #3 | Search: DE "Professional Competence" OR ((DE "Competence" OR TI competenc* OR AB competenc* OR TI capabilit* OR AB capabilit* OR DE "Competence" OR TI abilit* OR AB abilit*) OR ((TI knowledge OR AB knowledge) AND (TI skill* OR AB skill*)) |
| 4 | #4 | Search: TI assess* OR AB assess* OR DE "Measurement" OR TI measur* OR AB measur* OR DE "Evaluation" OR TI evaluat* OR AB evaluat* OR TI collec* OR AB collec* OR DE "Surveys" OR TI survey* OR AB survey* OR DE "Instrumentality" OR TI instrument* OR AB instrument* OR TI scale* OR AB scale* OR DE "Questionnaires" OR TI questionnaire* OR AB questionnaire* OR TI tool* OR AB tool* |
| 5 | #5 | Search：cl(“Psychometrics & Statistics & ‎Methodology” OR “Research ‎Methods & Experimental Design”) ‎OR (psychometr* OR clinimetr* OR ‎clinometr* OR “outcome ‎assessment” OR “outcome ‎measure*‎” OR ‎‎”observer variation” ‎OR reproducib* OR reliab*‎ OR ‎unreliab* OR valid* OR coefficient ‎OR ‎homogeneity OR homogeneous ‎‎OR ‎‎“internal consistency” OR ‎agreement OR precision OR ‎imprecision ‎OR “precise ‎values” ‎OR test-retest OR reliab* OR ‎stcompetence OR interrater OR inter-rater ‎‎OR intrarater OR ‎intra-rater OR ‎intertester OR inter-‎tester OR ‎intratester ‎OR intra-tester OR ‎interobserver OR inter-‎observer OR ‎intraobserver ‎OR intra-‎observer OR ‎intertechnician OR inter-technician ‎OR intratechnician ‎OR intra-‎technician ‎OR interexaminer OR ‎inter-examiner OR intraexaminer ‎OR intra-examiner OR ‎interassay ‎OR ‎inter-assay OR intraassay OR ‎intra-assay OR ‎interindividual OR ‎inter-individual ‎OR ‎intraindividual ‎OR intra-individual OR ‎interparticipant OR inter-participant ‎OR intraparticipant ‎OR ‎intra-‎participant OR ‎kappa OR kappa’s ‎OR kappas OR repeatab*‎ OR ‎generaliza* OR generalisa* OR ‎concordance OR discriminative OR ‎‎“known group” OR “factor analys*” ‎OR dimension* OR ‎subscale* ‎OR “‎item discriminant” ‎OR “interscale ‎correlation*” OR error* OR “‎individual varicompetence” OR “standard ‎error of ‎measurement” OR sensitiv* ‎OR responsive* OR “meaningful ‎change” OR ‎‎“ceiling effect” OR ‎‎“‎floor effect” OR “Item response ‎model” OR IRT OR Rasch OR “‎Differential ‎item functioning” OR ‎DIF OR ‎‎“computer adaptive testing‎” OR “item bank” OR “cross-cultural ‎‎equivalence”‎) OR (‎“cronbach* ‎alpha*” OR “replicab* measure*” ‎OR “replicab* finding*” OR “‎replicab* ‎result*” OR ‎‎“replicab* ‎test*” OR “repeated measure*” OR ‎‎“repeated finding*” OR “repeated ‎result*” OR ‎‎“repeated test*” OR “‎item correlation*” ‎OR “item ‎selection*” OR “item reduction*” ‎OR “Test retest” ‎OR “intraclass ‎correlation*” OR “multitrait scaling ‎analys*” OR “uncertainty measur*” ‎OR “varicompetence ‎analys*” OR “‎varicompetence value*” OR “minimal* ‎important change” OR “minimal* ‎important difference” ‎OR “minimal* ‎significant change” OR “minimal* ‎significant difference” OR “minimal* ‎detectable ‎change” OR “minimal* ‎detectable difference” OR “clinical* ‎important change” OR “clinical* ‎important ‎difference” OR “clinical* ‎significant change” OR “clinical* ‎significant difference” OR “clinical* ‎detectable ‎change” OR “clinical* ‎detectable difference” OR “small* ‎real change” OR “small* real ‎difference” OR ‎‎“small* detectable ‎change” OR “small* detectable ‎difference”‎) OR ‎‎(SU.EXACT.EXPLODE(“Measure‎ment”) OR ‎SU.EXACT.EXPLODE(“Error ‎Analysis”) OR ‎SU.EXACT.EXPLODE(“Test ‎Construction”) OR ‎SU.EXACT.EXPLODE(“Interrater ‎Relicompetence”) OR ‎SU.EXACT.EXPLODE(“Content ‎Analysis”) OR ‎SU.EXACT.EXPLODE(“Error of ‎Measurement”) OR ‎SU.EXACT.EXPLODE(“Factor ‎Structure”) OR ‎SU.EXACT.EXPLODE(“Testing ‎Methods”) OR ‎SU.EXACT.EXPLODE(“Statistical ‎Relicompetence”) OR ‎SU.EXACT.EXPLODE(“Consistency (Measurement)”) OR ‎SU.EXACT.EXPLODE(“Computer ‎Assisted Testing”) OR ‎SU.EXACT.EXPLODE(“Factor ‎Analysis”) OR ‎SU.EXACT.EXPLODE(“Prediction”‎‎) OR ‎SU.EXACT.EXPLODE(“Statistical ‎Validity”) OR ‎SU.EXACT.EXPLODE(“Prediction ‎Errors”))‎ |
| 6 | #6 | #1 AND #2 AND #3 AND #4 AND #5 |

**Table S10.** Search strategy for ERIC

| 1 | #1 | Search: title:nurs* |
| --- | --- | --- |
| 2 | #2 | Search: title:research |
| 3 | #3 | Search: (title:“professional competenc*” OR abstract:“professional competenc*”) OR ((title:“competenc*”OR abstract:“competenc*” OR title:“capabilit^*^” OR abstract:“capabilit^*^” OR title:“capacit*”OR abstract:“capacit*” OR abstract:“abilit*” OR title:“abilit*”) OR ((title:“knowledge” OR abstract:“knowledge”) AND (title:“skill*” OR abstract:“skill*”))) |
| 4 | #4 | Search: title:“assess*” OR abstract:“assess*” OR title:“measur*” OR abstract:“measur*” OR title:“evaluat*” OR abstract:“evaluat*” OR title:“collec*” OR abstract:“collec*” OR title:“survey*” OR abstract:“survey*” OR abstract:“instrument*” OR title:“instrument*” OR abstract:“scale*” OR title:“scale*” OR title:“questionnaire*” OR abstract:“questionnaire*” OR title:“tool*” OR abstract:“tool*” |
| 5 | #5 | Search: (descriptor:"Instrumentation" OR descriptor:"Methods" OR PUBLICATION TYPE:“Validation Study” OR PUBLICATION TYPE:“Comparative Study” OR descriptor:"Psychometrics" OR title:“psychometr*” OR abstract:“psychometr*” OR “clinimetr*” OR “clinometr*” OR title:“outcome assessment, health care” OR abstract:“outcome assessment, health care” OR title:“outcome assessment” OR abstract:“outcome assessment” OR “outcome measure*” OR title:“observer variation” OR abstract:“observer variation” OR title:“Health Status Indicators” OR abstract:“Health Status Indicators” OR title:“reproducibility of results” OR abstract:“reproducibility of results” OR title:“reproducib*” OR abstract:“reproducib*” OR descriptor:"Discriminant Analysis" OR title:“reliab*” OR abstract:“reliab*” OR title:“unreliab*” OR abstract:“unreliab*” OR title:“valid*” OR abstract:“valid*” OR title:“coefficient of variation” OR abstract:“coefficient of variation” OR title:“coefficient” OR abstract:“coefficient” OR title:“homogeneity” OR abstract:“homogeneity” OR title:“homogeneous” OR abstract:“homogeneous” OR title:“internal consistency” OR abstract:“internal consistency” OR ((title:“cronbach*” OR abstract:“cronbach*”) AND (title:“alpha” OR abstract:“alpha” OR title:“alphas” OR abstract:“alphas”)) OR ((title:“item” OR abstract:“item”) AND (title:“correlation*” OR abstract:“correlation*” OR title:“selection*” OR abstract:“selection*” OR title:“reduction*” OR abstract:“reduction*”)) OR “agreement” OR “precision” OR “imprecision” OR “precise values” OR title:“test-retest” OR abstract:“test-retest” OR ((descriptor:"Tests" OR title:“test” OR abstract:“test”) AND (title:“retest” OR abstract:“retest”)) OR ((title:“reliab*” OR abstract:“reliab*”) AND ((descriptor:"Tests" OR title:“test” OR abstract:“test”) OR (title:“retest” OR abstract:“retest”))) OR title:“stcompetence” OR abstract:“stcompetence” OR title:“interrater” OR abstract:“interrater” OR title:“inter-rater” OR abstract:“inter-rater” OR title:“intrarater” OR abstract:“intrarater” OR title:“intra-rater” OR abstract:“intra-rater” OR title:“intertester” OR abstract:“intertester” OR title:“inter-tester” OR abstract:“inter-tester” OR title:“intratester” OR abstract:“intratester” OR title:“intra-tester” OR abstract:“intra-tester” OR title:“interobserver” OR abstract:“interobserver” OR title:“inter-observer” OR abstract:“inter-observer” OR title:“intraobserver” OR abstract:“intraobserver” OR title:“intra-observer” OR abstract:“intra-observer” OR title:“intertechnician” OR abstract:“intertechnician” OR title:“inter-technician” OR abstract:“inter-technician” OR title:“intratechnician” OR abstract:“intratechnician” OR title:“intra-technician” OR abstract:“intra-technician” OR title:“interexaminer” OR abstract:“interexaminer” OR title:“inter-examiner” OR abstract:“inter-examiner” OR title:“intraexaminer” OR abstract:“intraexaminer” OR title:“intra-examiner” OR abstract:“intra-examiner” OR title:“interassay” OR abstract:“interassay” OR title:“inter-assay” OR abstract:“inter-assay” OR title:“intraassay” OR abstract:“intraassay” OR title:“intra-assay” OR abstract:“intra-assay” OR title:“interindividual” OR abstract:“interindividual” OR title:“inter-individual” OR abstract:“inter-individual” OR title:“intraindividual” OR abstract:“intraindividual” OR title:“intra-individual” OR abstract:“intra-individual” OR title:“interparticipant” OR abstract:“interparticipant” OR title:“inter-participant” OR abstract:“inter-participant” OR title:“intraparticipant” OR abstract:“intraparticipant” OR title:“intra-participant” OR abstract:“intra-participant” OR title:“kappa” OR abstract:“kappa” OR title:“kappa’s” OR abstract:“kappa’s” OR title:“kappas” OR abstract:“kappas” OR “repeatab*” OR ((“replicab*” OR “repeated”) AND (“measure” OR “measures” OR “findings” OR “result” OR “results” OR “test” OR “tests”)) OR title:“generaliza*” OR abstract:“generaliza*” OR title:“generalisa*” OR abstract:“generalisa*” OR title:“concordance” OR abstract:“concordance” OR ((title:“intraclass” OR abstract:“intraclass”) AND (title:“correlation*” OR abstract:“correlation*”)) OR title:“discriminative” OR abstract:“discriminative” OR title:“known group” OR abstract:“known group” OR descriptor:"Factor Analysis" OR title:“factor analysis” OR abstract:“factor analysis” OR title:“factor analyses” OR abstract:“factor analyses” OR descriptor:"Factor Structure" OR title:“factor structure” OR abstract:“factor structure” OR title:“factor structures” OR abstract:“factor structures” OR title:“dimension*” OR abstract:“dimension*” OR title:“subscale*” OR abstract:“subscale*” OR ((title:“multitrait” OR abstract:“multitrait”) AND (title:“scaling” OR abstract:“scaling”) AND (title:“analysis” OR abstract:“analysis” OR title:“analyses” OR abstract:“analyses”)) OR title:“item discriminant” OR abstract:“item discriminant” OR title:“interscale correlation*” OR abstract:“interscale correlation*” OR title:“error” OR abstract:“error” OR title:“errors” OR abstract:“errors” OR title:“individual varicompetence” OR abstract:“individual varicompetence” OR title:“interval varicompetence” OR abstract:“interval varicompetence” OR title:“rate varicompetence” OR abstract:“rate varicompetence” OR ((title:“varicompetence” OR abstract:“varicompetence”) AND (title:“analysis” OR abstract:“analysis” OR title:“values” OR abstract:“values”)) OR ((title:“uncertainty” OR abstract:“uncertainty”) AND (descriptor:"Measurement" OR title:“measurement” OR abstract:“measurement” OR title:“measuring” OR abstract:“measuring”)) OR title:“standard error of measurement” OR abstract:“standard error of measurement” OR title:“sensitiv*” OR abstract:“sensitiv*” OR title:“responsive*” OR abstract:“responsive*” OR ((title:“limit” OR abstract:“limit”) AND (title:“detection” OR abstract:“detection”)) OR title:“minimal detectable concentration” OR abstract:“minimal detectable concentration” OR title:“interpretab*” OR abstract:“interpretab*” OR ((title:“minimal” OR abstract:“minimal” OR title:“minimally” OR abstract:“minimally” OR title:“clinical” OR abstract:“clinical” OR title:“clinically” OR abstract:“clinically”) AND (title:“important” OR abstract:“important” OR title:“significant” OR abstract:“significant” OR title:“detectable” OR abstract:“detectable”) AND (descriptor:"Change" OR title:“change” OR abstract:“change” OR descriptor:"Differences" OR title:“difference” OR abstract:“difference”)) OR ((title:“small*” OR abstract:“small*”) AND (title:“real” OR abstract:“real” OR title:“detectable” OR abstract:“detectable”) AND (descriptor:"Change" OR title:“change” OR abstract:“change” OR descriptor:"Differences" OR title:“difference” OR abstract:“difference”)) OR title:“meaningful change” OR abstract:“meaningful change” OR title:“ceiling effect” OR abstract:“ceiling effect” OR title:“floor effect” OR abstract:“floor effect” OR title:“Item response model” OR abstract:“Item response model” OR title:“IRT” OR abstract:“IRT” OR title:“Rasch” OR abstract:“Rasch” OR title:“Differential item functioning” OR abstract:“Differential item functioning” OR title:“DIF” OR abstract:“DIF” OR title:“computer adaptive testing” OR abstract:“computer adaptive testing” OR descriptor:"Item Banks" OR title:“item bank” OR abstract:“item bank” OR title:“cross-cultural equivalence” OR abstract:“cross-cultural equivalence”) |
| 6 | #6 | Search: (PUBLICATION TYPE:“Address” OR PUBLICATION TYPE:“biography” OR PUBLICATION TYPE:“case reports” OR PUBLICATION TYPE:“comment” OR PUBLICATION TYPE:“directory” OR PUBLICATION TYPE:“editorial” OR PUBLICATION TYPE:“festschrift” OR PUBLICATION TYPE:“interview” OR PUBLICATION TYPE:“Lecture” OR PUBLICATION TYPE:“legal case OR PUBLICATION TYPE:“legislation” OR PUBLICATION TYPE:“letter” OR PUBLICATION TYPE:“news” OR PUBLICATION TYPE:“newspaper article” OR PUBLICATION TYPE:“patient education handout” OR PUBLICATION TYPE:“popular work” OR PUBLICATION TYPE:“congress” OR PUBLICATION TYPE:“consensus development conference” OR PUBLICATION TYPE:“consensus development conference, nih” OR PUBLICATION TYPE:“practice guideline”) NOT (descriptor:"Animals" NOT (title:humans OR abstract:humans)) |
| 7 | #7 | #1 AND #2 AND #3 AND #4 AND #5 NOT #6 |

**Table S11.** Search strategy for ProQuest

| 1 | #1 | Search: ti(nurs* ) |
| --- | --- | --- |
| 2 | #2 | Search: ti(research) |
| 3 | #3 | Search: ti(professional competenc*) OR ab(professional competenc*) OR ((ti(competenc*) OR ab(competenc*) OR ti(capabilit^*^) OR ab(capabilit^*^) OR MAINSUBJECT.EXACT("Capacity") OR ti(capacit*) OR ab(capacit*) OR ti(abilit*) OR ab(abilit*)) OR MAINSUBJECT.EXACT("Knowledge") OR ti(knowledge) OR ab(knowledge) AND MAINSUBJECT.EXACT("Skills") OR ti(skill*) OR ab(skill*)) |
| 4 | #4 | Search: ti(assess*) OR ab(assess*) OR ti(measur*) OR ab(measur*) OR ti(evaluat*) OR ab(evaluat*) OR ti(collec*) OR ab(collec*) OR ti(survey*) OR ab(survey*) OR MAINSUBJECT.EXACT("Instruments") OR ti(instrument*) OR ab(instrument*) OR ti(scale*) OR ab(scale*) OR MAINSUBJECT.EXACT("Questionnaires") OR ti(questionnaire*) OR ab(questionnaire*) OR ti(tool*) OR ab(tool*) |
| 5 | #5 | Search: ‎ (ti(instrumentation) OR ab(instrumentation) OR ti(methods) OR ab(methods) OR pub(“Validation Study”) OR pub(“Comparative Study”) OR ti(psychometr*) OR ab(psychometr*) OR noft(clinimetr*) OR noft(clinometr*) OR ti(“outcome assessment, health care”) OR ab(“outcome assessment, health care”) OR ti(outcome assessment) OR ab(outcome assessment) OR noft(outcome measure*) OR ti(“observer variation”) OR ab(“observer variation”) OR ti(“Health Status Indicators”) OR ab(“Health Status Indicators”) OR ti(“reproducibility of results”) OR ab(“reproducibility of results”) OR ti(reproducib*) OR ab(reproducib*) OR mainsubject(“discriminant analysis”) OR ti(“discriminant analysis”) OR ab(“discriminant analysis”) OR ti(reliab*) OR ab(reliab*) OR ti(unreliab*) OR ab(unreliab*) OR ti(valid*) OR ab(valid*) OR ti(“coefficient of variation”) OR ab(“coefficient of variation”) OR ti(coefficient) OR ab(coefficient) OR ti(homogeneity) OR ab(homogeneity) OR ti(homogeneous) OR ab(homogeneous) OR ti(“internal consistency”) OR ab(“internal consistency”) OR (ti(cronbach*) OR ab(cronbach*) AND (ti(alpha) OR ab(alpha) OR ti(alphas) OR ab(alphas))) OR ((ti(item) OR ab(item)) AND (ti(correlation*) OR ab(correlation*) OR ti(selection*) OR ab(selection*) OR ti(reduction*) OR ab(reduction*))) OR noft(agreement) OR noft(precision) OR noft(imprecision) OR noft(“precise values”) OR ti(test-retest) OR ab(test-retest) OR ((ti(test) OR ab(test)) AND (ti(retest) OR ab(retest))) OR (ti(reliab*) OR ab(reliab*) AND (ti(test) OR ab(test) OR ti(retest) OR ab(retest))) OR ti(stcompetence) OR ab(stcompetence) OR ti(interrater) OR ab(interrater) OR ti(inter-rater) OR ab(inter-rater) OR ti(intrarater) OR ab(intrarater) OR ti(intra-rater) OR ab(intra-rater) OR ti(intertester) OR ab(intertester) OR ti(inter-tester) OR ab(inter-tester) OR ti(intratester) OR ab(intratester) OR ti(intra-tester) OR ab(intra-tester) OR ti(interobserver) OR ab(interobserver) OR ti(inter-observer) OR ab(inter-observer) OR ti(intraobserver) OR ab(intraobserver) OR ti(intra-observer) OR ab(intra-observer) OR ti(intertechnician) OR ab(intertechnician) OR ti(inter-technician) OR ab(inter-technician) OR ti(intratechnician) OR ab(intratechnician) OR ti(intra-technician) OR ab(intra-technician) OR ti(interexaminer) OR ab(interexaminer) OR ti(inter-examiner) OR ab(inter-examiner) OR ti(intraexaminer) OR ab(intraexaminer) OR ti(intra-examiner) OR ab(intra-examiner) OR ti(interassay) OR ab(interassay) OR ti(inter-assay) OR ab(inter-assay) ti(intraassay) OR ab(intraassay) OR ti(intra-assay) OR ab(intra-assay) OR ti(interindividual) OR ab(interindividual) OR ti(inter-individual) OR ab(inter-individual) OR ti(intraindividual) OR ab(intraindividual) OR ti(intra-individual) OR ab(intra-individual) OR ti(interparticipant) OR ab(interparticipant) OR ti(inter-participant) OR ab(inter-participant) OR ti(intraparticipant) OR ab(intraparticipant) OR ti(intra-participant) OR ab(intra-participant) OR ti(kappa) OR ab(kappa) OR ti(kappa’s) OR ab(kappa’s) OR ti(kappas) OR ab(kappas) OR noft (repeatab*) OR (noft(replicab*) OR noft(repeated)) AND (noft(measure) OR noft(measures) OR noft(findings) OR noft(result) OR noft(results) OR noft(test) OR noft(tests))) OR ti(generaliza*) OR ab(generaliza*) OR ti(generalisa*) OR ab(generalisa*) OR ti(concordance) OR ab(concordance) OR ((ti(intraclass) OR ab(intraclass)) AND (ti(correlation*) OR ab(correlation*))) OR ti(discriminative) OR ab(discriminative) OR ti(“known group”) OR ab(“known group”) OR ti(“factor analysis”) OR ab(“factor analysis”) OR ti(“factor analyses”) OR ab(“factor analyses”) OR ti(“factor structure”) OR ab(“factor structure”) OR ti(“factor structures”) OR ab(“factor structures”) OR ti(dimension*) OR ab(dimension*) OR ti(subscale*) OR ab(subscale*) OR ((ti(multitrait) OR ab(multitrait)) AND (ti(scaling) OR ab(scaling)) OR ((ti(analysis) OR ab(analysis)) OR (ti(analyses) OR ab(analyses))) OR ti(“item discriminant”) OR ab(“item discriminant”) OR ti(“interscale correlation*”) OR ab(“interscale correlation*”) OR ti(error) OR ab(error) OR ti(errors) OR ab(errors) OR ti(“individual varicompetence”) OR ab(“individual varicompetence”) OR ti(“interval varicompetence”) OR ab(“interval varicompetence”) OR ti(“rate varicompetence”) OR ab(“rate varicompetence”) OR ((ti(varicompetence) OR ab(varicompetence)) AND (ti(analysis) OR ab(analysis) OR ti(values) OR ab(values))) OR (((ti(uncertainty) OR ab(uncertainty)) AND ti(measurement) OR ab(measurement) OR ti(measuring) OR ab(measuring))) OR ti(“standard error of measurement”) OR ab(“standard error of measurement”) OR ti(sensitiv*) OR ab(sensitiv*) OR ti(responsive*) OR ab(responsive*) OR ((ti(limit) OR ab(limit)) AND (ti(detection) OR ab(detection))) OR ti(“minimal detectable concentration”) OR ab(“minimal detectable concentration”) OR ti(interpretab*) OR ab(interpretab*) OR ((ti(minimal) OR ab(minimal) OR ti(minimally) OR ab(minimally) OR ti(clinical) OR ab(clinical) OR ti(clinically) OR ab(clinically)) AND (ti(important) OR ab(important) OR ti(significant) OR ab(significant) OR ti(detectable) OR ab(detectable)) AND (ti(change) OR ab(change) OR ti(difference) OR ab(difference))) OR ((ti(small*) OR ab(small*)) AND (ti(real) OR ab(real) OR ti(detectable) OR ab(detectable)) AND (ti(change) OR ab(change) OR ti(difference) OR ab(difference))) OR ti(“meaningful change”) OR ab(“meaningful change”) OR ti(“ceiling effect”) OR ab(“ceiling effect”) OR ti(“floor effect”) OR ab(“floor effect”) OR ti(“Item response model”) OR ab(“Item response model”) OR ti(IRT) OR ab(IRT) OR ti(Rasch) OR ab(Rasch) OR ti(“Differential item functioning”) OR ab(“Differential item functioning”) OR ti(DIF) OR ab(DIF) OR ti(“computer adaptive testing”) OR ab(“computer adaptive testing”) OR ti(“item bank”) OR ab(“item bank”) OR ti(“cross-cultural equivalence”) OR ab(“cross-cultural equivalence”)) |
| 6 | #6 | #1 AND #2 AND #3 AND #4 AND #5 |

**Table S12.** Search strategy for Wanfang

| 1 | #1 | TI = nursing OR TI = nurses OR TI= nursing students |
| --- | --- | --- |
| 2 | #2 | SU = scientific research competence OR SU = scientific research knowledge OR SU = scientific nursing skills OR SU = research competence OR SU = research knowledge OR SU = nursing skills |
| 3 | #3 | SU = evaluate OR SU = measure OR SU = collect OR SU = survey OR SU = instruments OR SU = questionnaires OR SU = scales |
| 4 | #4 | #1 AND #2 AND #3 |

**Table S13.** Search strategy for CNKI

| 1 | #1 | TI = nursing OR TI = nurses OR TI= nursing students |
| --- | --- | --- |
| 2 | #2 | SU = scientific research competence OR SU = scientific research knowledge OR SU = scientific nursing skills OR SU = research competence OR SU = research knowledge OR SU = nursing skills |
| 3 | #3 | SU = evaluate OR SU = measure OR SU = collect OR SU = survey OR SU = instruments OR SU = questionnaires OR SU = scales |
| 4 | #4 | #1 AND #2 AND #3 |

Note: Wanfang and CNKI are both Chinese databases, the Chinese retrieval strategy was used for the retrieval of these two databases.
